# Phylogenetic and Proteomic Analyses of Segment 2 Sequence Reveals the Presence of Two Variants of a Divergent Amnoonvirus (Family: *Amnoonviridae*) Infecting Nile Tilapia (*Oreochromis niloticus*)

**DOI:** 10.3390/microorganisms14020343

**Published:** 2026-02-02

**Authors:** Amel M. El Asely, Mohamed Shawky Khalifa, Wei Xu, Adel A. Shaheen, Mohamed Faisal

**Affiliations:** 1 Department of Aquatic Animal Medicine, Faculty of Veterinary Medicine, Benha University, Toukh 13736, Egypt; amlvet@yahoo.com (A.M.E.A.); shaheen_aa@yahoo.com (A.A.S.); 2 Department of Fisheries and Wildlife, College of Agriculture and Natural Resources, Michigan State University, East Lansing, MI 48824, USA; khalifa9@msu.edu; 3 Department of Veterinary Physiology & Pharmacology, Texas A&M University College of Veterinary and Biomedical Sciences, College Station, TX 77843, USA; wxu1@tamu.edu; 4 Department of Pathobiology and Diagnostic Investigation, College of Veterinary Medicine, Michigan State University, East Lansing, MI 48824, USA

**Keywords:** *Amnoonviridae*, polymerase basic subunit 2, divergence, mutations, epitopes, Illumina sequencing

## Abstract

Illumina sequencing of segment 2, which encodes the polymerase basic subunit 2 (PB2) of the RNA-dependent RNA polymerase of a divergent amnoonvirus recently detected in tissues of Nile tilapia farmed in Egypt, revealed the presence of two genetic variants of the same virus: AmnoonvirusEGY1F and -H. The phylogenetic and genetic analyses presented in this study support the inclusion of both variants in the genus *Tilapinevirus*, family *Amnoonviridae*, order *Articulavirales*. The Egyptian strains formed distinct, well-supported clades in both nucleotide- and amino acid-based trees, showing a notable divergence from unclassified amnoonviruses and clustered with members of the genus *Tilapinevirus*. Within the genus *Tilapinevirus*, both Egyptian strains were divergent from all tilapia lake virus (TiLV) strains, whose full RNA segment 2 sequences are available in public databases, as well as the newly isolated *Tilapinevirus poikilos* from the fancy-tailed guppy. The Egyptian strains were also divergent from TiLV strains identified in Israel and Lake Victoria. Although the PB2 proteins of AmnoonvirusEGY1F and -H exhibited striking similarity, several mutations were detected that altered the sequence of their antigenic cell epitopes. Some of these mutations in the AmnoonvirusEGY1H strain were predicted to affect PB2-encoded functions. Collectively, findings of this study aid in the growing understanding of viral diversity and PB2 evolution in emerging amnoonviruses, particularly the role of amino acid substitutions in affecting the encoded protein structure, function, and immunogenicity.

## 1. Introduction

In 2006, an RNA virus emerged and was associated with massive mortality episodes in tilapine fish species in Lake Kinneret in the Jordan Rift Valley that was later named the tilapia lake virus (TiLV) [1]. The unique biological properties of TiLV made its placement in the proper taxonomic classification of viruses a daunting task. On the one hand, TiLV has some resemblance to members of the family *Orthomyxoviridae*, having a single- stranded, negative-sense, segmented RNA, with its RNA-dependent RNA polymerase encoded by the first three RNA segments bearing weak sequence homology to that of other orthomyxoviruses [2]. On the other hand, this decasegmented RNA TiLV exhibited unique properties that are different from other orthomyxoviruses [3,4,5]. To solve this taxonomy uncertainty, TiLV was placed in a new family, the *Amnoonviridae*, which together with the family *Orthomyxoviridae* formed the order *Articulavirales* [6,7]. Within the family *Amnoonviridae*, TiLV was placed as a single member of the family’s single genus, the *Tilapinevirus*, and the species *T. tilapiae* [7]. Since then, several studies have proposed other viruses to be added to the *Amnoonviridae* family [8,9,10,11].

In a previous study using the Oxford Nanopore sequencing platform, we detected a divergent amnoonvirus in the virome of farmed Nile tilapia in Egypt (referred to as AmnoonvirusEGY1) [12]. Phylogenetic analyses performed on partial sequences of segments 1, 5, and 7, placed AmnoonvirusEGY1 within the genus *Tilapinevirus* in the family *Amnoonviridae*. This virus, however, exhibited clear genetic divergence from the other two members in the genus: *T. tilapiae* (TiLV) and *T. poikilos*. Moreover, further phylogenetic analyses confirmed AmnoonvirusEGY1 as being distinct from TiLV strains detected in the neighboring Israel and whose sequences of segments 1, 5, and 7 are available in public databases [12]. In Egypt, TiLV was detected in farmed Nile tilapia associated with mortality episodes [13,14] and was also distinct from other TiLV strains reported from Israel based on comparisons with partial sequences of segments 3, 4, and 9 [14]. Around the same time, Mugimba et al. [15] reported TiLV in wild and farmed tilapia from Lake Victoria in Africa, which is a part of the River Nile basin, in which the Egyptian farms, where AmnoonvirusEGY1 was detected, lie. Although Mugimba et al. [15] designed primers based on published TiLV sequences of the 10 segments, only partial segment 2 sequences of Lake Victoria viruses are available in public databases. How related the TiLV strains previously reported in Egypt are to those detected in Lake Victoria and to the recently detected AmnoonvirusEGY1 is currently unknown mainly because of the inconsistent availability of African TiLV nucleotide sequences of each segment.

Most of the information available on the RNA-dependent RNA polymerase (RdRp) and its components in members of the order *Articulavirales* comes from studies performed on influenza viruses [16]. Amnoonviruses’ RdRp consists of three subunits: subunit 1 (polymerase basic 1, PB1), subunit 2 (polymerase basic 2, PB2), and subunit 3 (polymerase acidic, PA), encoded by segments 1–3, respectively [2,4]. Indeed, phylogenetic analyses of PB2 of several orthomyxoviruses have provided evidence on lineage divergence as the leading driver for the appearance of novel viruses or sublineages and the mechanisms by which the virus increases its pathogenicity and ability to evade host defense mechanisms [17,18,19]. One small and one relatively large open reading frame (ORF) exist in TiLV-segment 2 (1471 bp), with the larger ORF encoding the 457 amino acids of the modular PB2 protein [4]. The TiLV-PB2 protein structure exhibits distinct domains such as the N-terminal domain and several C-domains [20].

To this end, the first objective of this study was to sequence the full length of segment 2 including the RNA polymerase subunit 2 (PB2) gene of the AmnoonvirusEGY1 detected earlier in tissues of apparently healthy Nile tilapia reared in two farms in Egypt and to compare its generated full length segment 2 sequences to publicly available sequences of other TiLV strains, particularly those detected in regional sites such as Israel and Lake Victoria. The second aim of this study was to determine if the divergence of AmnoonvirusEGY1 is associated with changes in the structure and antigenicity of the PB2 protein. Information to be generated from this research is important to further characterize a divergent amnoonvirus strain that is infecting Nile tilapia, one of the most important fish species for aquaculture worldwide.

## 2. Material and Methods

### 2.1. Nile Tilapia-AmnoonvirusEGY1 Infected Samples

Pooled tissue homogenate samples, originally collected from Nile tilapia (*Oreochromis niloticus*), were made available for this study by Ezzat et al. [12]. The fish originated from two farms: Bahr Yusef Farm F (29°18′27.855″ N and 30°50′45.447″ E) in Faiyum Governorate and Farm H in Elhusseiniya District (31°3′30.481″ N and 32°6′20.346″ E), Sharqia Governorate, Egypt. Additional information on fish samples and collection sites can be found in Ezzat et al. [12]. Originally, the fish were collected, handled, and euthanized according to the protocols approved by the Institutional Animal Care and Use Committee Research Ethics Board, Faculty of Veterinary Medicine, Benha University, Egypt (Ethical Number: BUFVTM56-11-23). Tissue pools used in this study were from Nile tilapia infected with the divergent AmnoonvirusEGY1 [12]. Tissue samples were immersed in RNAlater^TM^ (Thermo Fisher Scientific, Waltham, MA, USA) and stored at −80 °C until processing.

### 2.2. RNA Extraction and cDNA Synthesis

Total RNA from 50 mg of pooled tissue homogenate was extracted using the RNeasy Mini Kit (Qiagen, Hilden, Germany) according to the manufacturer’s instructions. Eluted RNA samples were kept at −80 °C until use. The quantification of the extracted RNA was conducted with the Qubit™ RNA BR Assay Kit (Invitrogen, Carlsbad, CA, USA). RNA samples were reverse transcribed into cDNA using the Illumina NEBNext XT DNA preparation kit (New England Biolabs, Ipswich, MA, USA) according to the manufacturer’s instructions. cDNA samples were then quantified using the Qubit™ dsDNA High Sensitivity Assay Kit (Invitrogen) and stored at −20 °C until further processing.

### 2.3. Illumina Sequencing

The barcoding and sequencing of cDNA libraries were performed using the Nextera CD Indexes (Illumina, San Diego, CA, USA). Purified libraries were normalized to 4 nM, pooled, denatured using 0.2 N sodium acetate, diluted to a final concentration of 8 pM, and spiked with 1% PhiX Control v3 (Illumina). Sequencing was performed using a 600-cycle v3 MiSeq Reagent Kit on the Illumina MiSeq instrument (Illumina) using sequencing parameters of 150 bp (paired ends) and a Q30 value of sequencing > 83%. Fastq files produced from Illumina MiSeq were analyzed using CLC Workbench (Qiagen) to create FASTA files. Low-quality reads were then filtered and the adapter sequences trimmed with Trimmomatic (GitHub, San Francisco, CA, USA). Base calling was performed using Illumina software v2.1.7 to convert raw data from the sequencer into sequence reads with the low-quality reads removed and adaptor sequencing trimmed in CLC Genomics Workbench v10.1.1 (Qiagen) using default parameters. In parallel, de novo assembly was performed using SPAdes v3.10.0 (GitHub), and the resulting RNA segment 2 contigs were compared to the reference-guided consensus sequence generated using the published RNA segment 2 reference genome (NC_029921.1) to confirm sequence integrity. No major discrepancies were observed between approaches, and the reference-guided consensus sequence was used for downstream analyses.

### 2.4. Phylogenetic and Genetic Analyses

Two contigs were assembled, each originating from a different farm and hereafter referred to as AmnoonvirusEGY1F and AmnoonvirusEGY1H. Alignment of the full-length segment 2 nucleotide sequences of the two contigs “GenBank accession number PX458600 for AmnoonvirusEGY1F and PX458601 for AmnoonvirusEGY1H” revealed nucleotide differences at multiple positions (Figure S1). The two contigs were further compared using the NCBI BLASTn (Align Sequences Nucleotide BLAST) v2.17.0 tool which showed similarity to members of the order *Articulavirales*. To confirm the evolutionary relationships of AmnoonvirusEGY1F and -H with other members of the order *Articulavirales*, the full-length segment 2 sequences of both contigs were aligned with segment 2 nucleotide sequences from amnoonviruses and with segment 1 of orthomyxoviruses, which is functionally equivalent to segment 2 in amnoonviruses (Table 1).

Further analysis focused on finding the taxonomic position of the Egyptian strains within the family *Amnoonviridae*. For this purpose, segment 2 sequences from five unclassified amnoonviruses [10], in addition to the ICTV-index tilapia lake virus (TiLV) strain [1], the TiLV-like Maracas-2015-1 strain from guppies [21], and the fancy-tailed guppy virus (FTGV) Cobra-B2 strain [9], were aligned with those of AmnoonvirusEGY1F and -H and analyzed. An additional, broader analysis was performed focusing on viruses that are, or proposed by the ICTV to be, in the genus *Tilapinevirus*. This analysis incorporated segment 2 full-length sequences from 29 TiLV isolates, two TiLV-like guppy strains (Maracas-2015-1 and Maracas-2015-2), and two fancy-tailed guppy (FTGV) strains. Full-segment 1 sequence of the orthomyxovirus infectious salmon anemia virus (ISAV) was included as an outgroup to root the phylogenetic tree. Because the only gene sequence available in public databases from TiLV strains isolated from the River Nile basin in Africa are partial segment 2 sequences [15], a fourth phylogenetic analysis was performed using publicly available partial segment 2 sequences to determine the similarity between the African strains and those being characterized in this study.

All sequences were aligned using the MUSCLE algorithm implemented in MEGA v12.0.11 [22]. Phylogenetic trees were constructed using the maximum likelihood (ML) method, applying the best-fit nucleotide substitution model as determined by MEGA. Tree reliability was assessed via 1000 non-parametric bootstrap replicates. Pairwise nucleotide and amino acid distances were calculated using the p-distance model. To complement the nucleotide-level analysis, a protein-level phylogenetic assessment was performed using the same virus group described above. Nucleotide sequences were translated into amino acid sequences using the standard genetic code in MEGA. The resulting protein sequences were aligned with MUSCLE, and ML trees were generated using the Jones–Taylor–Thornton (JTT) model, a widely used substitution model for protein evolution. Bootstrap support was evaluated with 1000 replicates. This analysis provided additional insights into the evolutionary divergence and potential functional conservation of segment 2-encoded proteins of the two strains being characterized in this study.

### 2.5. Predicting the Three-Dimensional (3D) PB2 Protein Structure of the Divergent AmnoonvirusEGY1 Variants

The PB2 open reading frame-encoded amino acid sequences predicted from AmnoonvirusEGY1F and -H segment 2 sequences were used for 3D model generation. The start of the PB2 protein sequence was the first methionine (Met) from the 5′ end. Protein sequences from the two Egyptian viruses were aligned along with the PB2 proteins of TiLV and their 3D structures predicted using the SWISS-MODEL Workspace updtaed in 2025 [23] and visualized by Swiss-Pdb Viewer v4.1 [24]. The two 3D structures of the Egyptian strains were overlaid onto the 3D structure of the TiLV index strain polymerase subunit 2 as predicted during the pre-initiation of the transcriptase conformation (https://www.ncbi.nlm.nih.gov/Structure/pdb/8PT2, accessed on 18 September 2025) [20]. To evaluate the integrity and stereochemical quality of the predicted PB2 3D structures, the MolProbity software version 4.4 (https://proteiniq.io/app/molprobity, accessed on 18 September 2025) was employed. This valuable tool collates multifaceted computational analyses to check for the overall geometric correctness of the predicted 3D protein structure and combine them into one value: the MolProbity score. This score is the resultant of three values: the clash score which measures steric overlaps between atoms, the Ramachandran statistics that evaluate the soundness of the structural backbone and its dihedral angles, and the number of Rotamer outliers which reflects the soundness of sidechain conformations. The analysis also identifies the bonds and angles that deviate from ideal values and geometry.

A number of bioinformatic tools were used to predict if amino acid substitutions in the Egyptian divergent amnoonviruses can impact the PB2 protein structure and function. For comparative analysis, the RdRp PB2 amino acid sequence of the index TiLV [8PT6] [1,25] was used as the baseline for mutation mapping and structure-based analysis. As described above, amino acid substitutions in AmnoonvirusEGY1F and -H were identified by pairwise comparison against the TiLV index strain and recorded with the first letter of the reference residue followed by its position in the sequence and then the first letter of the substituted residue (e.g., A230E).

The sequence-based sorting-intolerant-from-tolerant model (SIFT) [26], was employed to predict the degree to which each mutation can potentially impact the protein function. A substitution is predicted to be either deleterious/intolerant if it exists in a position in the protein that is highly conserved or benign/tolerated if the change is predicted to be not disruptive to protein functions [26]. The degree of conservation at each residue position was assessed using the conservation-based ConSurf-style framework (https://vdclab-wiki.herokuapp.com/en/structure/evolutionary_conserved_residues/ConSurf, accessed on 18–23 January 2026). The ConSurf server (https://ConSurf.tau.ac.il, accesssed on 18–23 January 2026) aligned 35 PB2 protein sequences of homologous strains and estimated evolutionary conservation of each residue based on the phylogenetic relationship between homologous sequences. Each residue position was assigned to a conservation category: variable, intermediate, or highly conserved. Mutations occurring at highly conserved positions were interpreted as more likely to influence protein function. The structure-based FoldX-style Reasoning Server (https://foldxsuite.crg.eu/, accessed on 16–23 January 2026) was used to predict if each mutation could destabilize PB2 protein structure cores. The combination of the three predictions classifies a mutation as likely neutral, mildly impactful, or potentially significant.

**Table 1:** Viruses included in segment 2 phylogenetic analyses. The table includes virus family, name, geographic origin, host species, and references. It includes both classified and unclassified members of the family *Amnoonviridae*, including AmnoonvirusEGY1F and -H. Representative viruses from the family *Orthomyxoviridae* are also included to provide broader phylogenetic context. GenBank accession numbers are provided for sequence reference.

**Family**	**Virus Name**	**Geographical Location**	**Target Host and (Reservoir Host)**	**References**
* Amnoonviridae *	AmnoonvirusEGY1F	Egypt	Nile tilapia	PX458600, this study
*Amnoonviridae*	AmnoonvirusEGY1H	Egypt	Nile tilapia	PX458601, this study
*Amnoonviridae*	Tilapia lake virus	Israel	Tilapia	KU751815.1 [1]
*Amnoonviridae*	Asotus virus 1	Japan	Amur catfish, *Silurus asotus*	GHGF01027066.1 [10]
*Amnoonviridae*	Asotus virus 2	Japan	Amur catfish	GHGF01034639.1 [10]
*Amnoonviridae*	Przewalskii virus	China	Scaleless carp, *Gymnocypris przewalskii*	GHYJ01010906.1 [10]
*Amnoonviridae*	Stewartii virus	China	Cyprinid fish, *Oxygymnocypris stewartii*	GIBO01013027.1 [10]
*Amnoonviridae*	Namensis virus	China	Cyprinid fish, *Gymnocypris namensis*	GHYH01005036.1 [10]
*Amnoonviridae*	Tilapia lake virus-like virus	Caribbean	*Guppy* , *Poecilia reticulata*	BK063201.1 [21]
*Amnoonviridae*	Fancy-tailed guppy virus	USA	*Guppy*	PP409996.1 [9]
*Orthomyxoviridae*	Infectious salmon anemia virus	Norway	Atlantic salmon, *Salmo salar*	NC006505.1, [27] “Direct submission”
*Orthomyxoviridae*	Rainbow trout orthomyxovirus-1	USA	Rainbow trout, *Oncorhynchus mykiss*	KX882061.1 [28]
*Orthomyxoviridae*	Pilchard orthomyxovirus	Australia	Atlantic salmon, *Salmo salar*	NC078607.1 [29]
*Orthomyxoviridae*	Wuhan spiny eel influenza virus	China	Spiny eel, *Macrognathus aculeatus*	MG600037.1 [30]
*Orthomyxoviridae*	Wenling hagfish influenza virus	China	Wenling hagfish, *Eptatretus burgeri*	MG600052.1 [30]
*Orthomyxoviridae*	Wuhan Asiatic toad influenza virus	China	Asiatic toad, *Bufo gargarizans*	MG600046.1 [30]
*Orthomyxoviridae*	Influenza A virus	USA	Blue-winged teal, *Anas discors*	KJ413482.1 [31]
*Orthomyxoviridae*	Influenza B virus	Canada	Egg-grown virus	NC002205.1 [32]
*Orthomyxoviridae*	Influenza C virus	Japan	Human	NC006307.2 [33]
*Orthomyxoviridae*	Influenza D virus	France	Bovine	LN559120.1 [34]

### 2.6. Assessing the Predicted Antigenicity of AmnoonvirusEGY1F and -H PB2 Proteins

Epitopes determine the antigenicity of a virus, as they are recognized by the host immune system and thereby initiate an immune response designed to neutralize the invading virus and develop memory cells. An in silico computational analysis was performed to predict the antigenic epitopes in the PB2 proteins of AmnoonvirusEGY1F and -H and to determine how these epitopes are different from those of the PB2 protein of the TiLV index strain. In this study, we selected two types of epitopes that are of paramount importance for the immune system to recognize and combat the virus: the linear B-cells (LBL) and the cytotoxic T lymphocytes (CTL). In fish, CTLs recognize and lyse virus-infected cells [35], while LBL epitopes induce B-cell activation, leading to the production of high-affinity binding and neutralizing antibodies [36]. The TiLV index strain proteome (GenBank accession #AMR44594.1) was retrieved from the NCBI database. As mentioned above, coding sequences of AmnoonvirusEGY1F and -H, each matching the index strain in length, were translated to generate their respective segment 2 proteomes for antigenic characterization. CTL epitopes were analyzed using the NetCTL v1.2 server (https://services.healthtech.dtu.dk/services/NetCTL-1.2/, accessed on 16 August 2025) [37] under default parameters. LBL epitopes were predicted using the ABCpred server (https://webs.iiitd.edu.in/raghava/abcpred/ABC_submission.html, accessed on 16 August 2025) [38] with a modified threshold value of 0.75 to increase prediction specificity. The antigenicity of all predicted epitopes from both the CTL and LBL groups was subsequently assessed using the VaxiJen v2.0 server (https://www.ddg-pharmfac.net/vaxijen/VaxiJen/VaxiJen.html, accessed on 16 August 2025) [39] under default parameters with a threshold value of 0.4. VaxiJen 2.0 bases its antigenicity prediction on the physicochemical properties of proteins, without relying on sequence alignment. Epitopes are ranked by two scores: prediction score and antigenic score. The prediction score is a quantitative measure based on algorithms designed to predict the potential of a peptide or protein to be an epitope [40], while the antigenic score evaluates the likelihood of an epitope sequence to induce an immune response [40]. As defined by NetCTL, CTL epitope prediction ranking is based on the epitope C score which assesses the likelihood of a given peptide to bind to the major histocompatibility complex class I (MHC I) molecules, a step that is necessary for CTL response [37].

## 3. Results

### 3.1. Illumina Sequencing Output and Quality Metrics

Illumina sequencing resulted in two contigs constituting the full length of segment 2 extracted from tissue homogenates of tilapia from Farm F (contig AmnoonvirusEGY1F) and Farm H (contig AmnoonvirusEGY1H). AmnoonvirusEGY1F segment 2 sequencing yielded 2,428,980 total paired end reads (2 × 150 bp), of which 2,221,059 were high-quality reads (91.4%), with a mean read length of 117 bp (107 bp after quality filtering) and GC content of 46.39%. The AmnoonvirusEGY1H segment 2 sequencing displayed similar sequencing criteria, yielding 2,357,238 total reads, of which 95.1% were high quality reads. The mean read length of this run was 119.3 bp (113.5 bp after quality filtering), and the GC content was 47.1%. The Phred quality score of both sequences was ≥Q30, signifying an error rate of <0.1% as expected for Illumina MiSeq runs. The assembly for both segments yielded two 1470 bp contigs that were uniform in coverage with no gaps. The average coverage was 161,668 and 173,086 reads per nucleotide for AmnoonvirusEGY1F and -H, respectively. The integrity of the assembly was confirmed by mapping the reads back to the complete sequences using Bowtie 2 as detailed in Langmead and Salzberg [41] with default parameters. As mentioned above, the sequences of the two assembled segment 2 contigs of this study have been deposited in GenBank under the accession numbers PX458600 for AmnoonvirusEGY1F and PX458601 for AmoonvirusEGY1H.

### 3.2. Phylogenetic and Genetic Analyses

**Figure 1:**
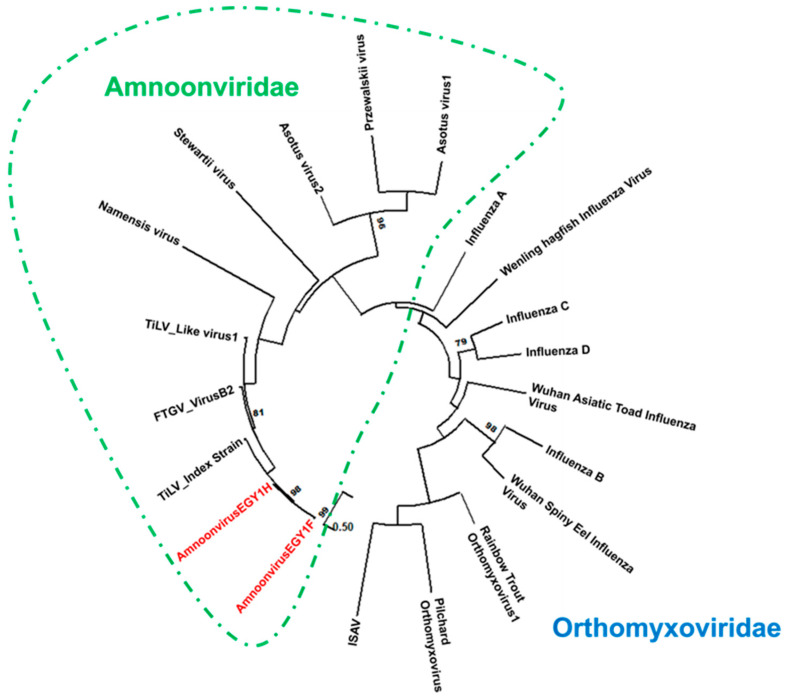
Circular maximum likelihood phylogenetic tree based on nucleotide sequences of the full-length segment that contains the PB2 gene (segment 2 in amnoonviruses and segment 1 in orthomyxoviruses), illustrating the evolutionary placement of the newly identified Egyptian amnoonviruses (AmnoonvirusEGY1F and -H, highlighted in red) within the order *Articulavirales*. The tree includes representative members of the family *Amnoonviridae*, including the tilapia lake virus index strain (TiLV), as well as several unclassified amnoonviruses (Asotus virus-1, Asotus virus-2, Stewartii virus, Przewalskii virus, and Namensis virus). Branch lengths represent the number of nucleotide substitutions per site, and bootstrap values ≥ 70% are shown at the nodes. The circular layout emphasizes the clustering of both AmnoonvirusEGY1s with other *Amnoonviridae* members and highlights its close phylogenetic affinity to both classified and unclassified viruses within the order.

The initial analysis was conducted by comparing the two contigs of the full-length segment 2 using pairwise nucleotide alignment. The comparison revealed a high level of sequence similarity, with 98% identity and no gaps detected across the 1470 bp length (Figure S1). There were 30 single-nucleotide polymorphisms (SNPs) between the two contigs, resulting in 28 amino acid substitutions in the translated amino acids. Despite the high sequence similarity of 98%, each contig was analyzed independently. Phylogenetic analysis of the full-length segment 2 sequences positioned both amnoonviruses of this study within a well-supported clade in the *Amnoonviridae* family (bootstrap value = 98%), clustering alongside the tilapia lake virus (TiLV) index strain and other amnoonviruses (Figure 1). The distinct clade formed by nucleotide sequences of the two strains of this study demonstrates a close evolutionary relationship with TiLV. On the contrary, the Egyptian variants were clearly divergent from other members of the *Orthomyxoviridae* family included in the analysis. This phylogenetic placement supports the classification of AmnoonvirusEGY1F and -H as genetic variants of one strain within the *Tilapinevirus* genus in the *Amnoonviridae* family. Further genetic analysis using the full-length segment 2 sequences of AmnoonvirusEGY1F and -H confirms the notable divergence from other *Articulavirales* members. Pairwise nucleotide distances ranged from 0.052 to 0.059 (vs. TiLV index strain) to 0.738 and 0.742 (vs. ISAV) (Table S1). Amino acid distances were generally lower, ranging from 0.010 and 0.020 (vs. TiLV index strain) to 0.951 for both (vs. influenza A), consistent with functional constraints on PB2 (Table S2).

**Figure 2:**
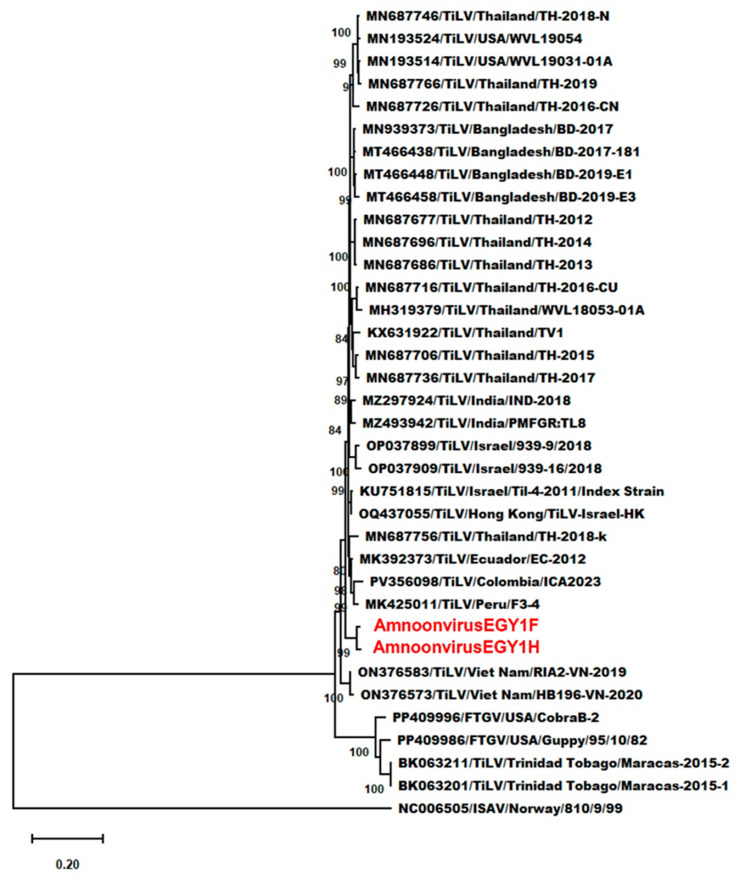
Maximum likelihood phylogram based on full-length segment 2 nucleotide sequences, illustrating the evolutionary relationships within the genus *Tilapinevirus*. The tree includes AmnoonvirusEGY1F and -H (shown in red), 29 TiLV isolates, Maracas-2015-1 and Maracas-2015-2, and two fancy-tailed guppy virus (FTGV) strains. Isolates are labeled with GenBank accession numbers, virus names, countries of origin, and isolate identifiers. Branch lengths represent the number of nucleotide substitutions per site, and bootstrap support values ≥ 70%.

**Figure 3:**
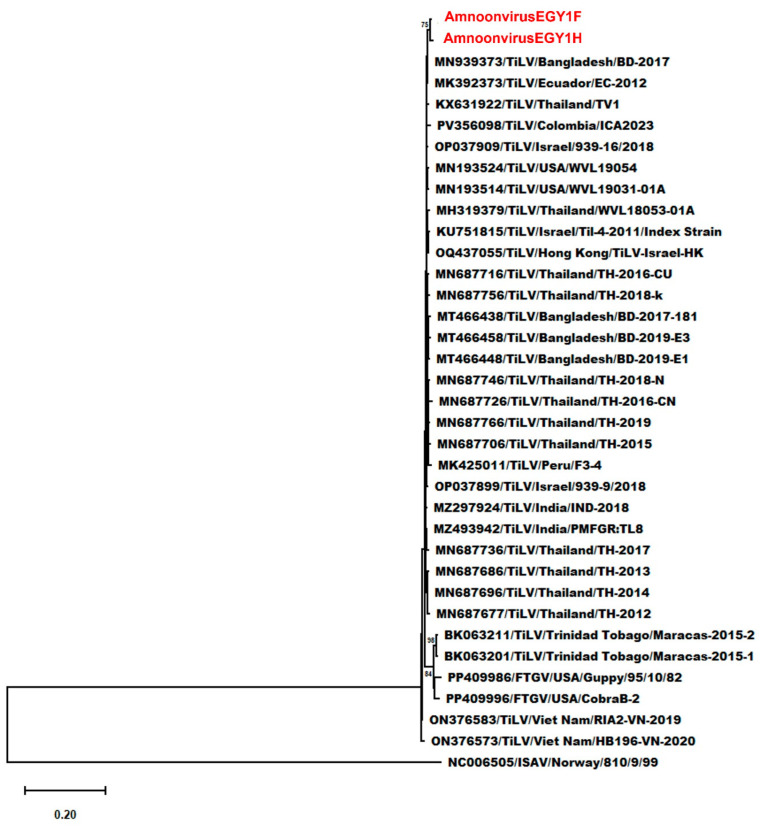
Maximum likelihood phylogenetic tree based on amino acid sequences of the segment 2 protein, illustrating evolutionary relationships within the family *Amnoonviridae.* The tree includes the newly identified Egyptian amnoonviruses (AmnoonvirusEGY1F and -H, shown in red), 29 tilapia lake virus (TiLV) isolates, two TiLV-like strains from guppies (Maracas-2015-1 and Maracas-2015-2), and two fancy-tailed guppy virus (FTGV) strains. The tree was constructed using the Jones–Taylor–Thornton (JTT) substitution model. Branch lengths represent the number of amino acid substitutions per site, and bootstrap support values ≥ 70% are shown at the nodes.

Within the genus *Tilapinevirus*, detailed genetic analyses using complete segment 2 sequences from TiLV, TiLV-like strains, and FTGV isolates, with the segment 1 sequence of ISAV as an outgroup, showed that AmnoonvirusEGY1F and -H formed a distinct, well-supported clade among TiLV isolates from various regions and hosts. This clade exhibited a strong support (bootstrap = 100%), indicating clear genetic divergence from other published TiLV strain sequences (Figure 2). Nucleotide sequence differences ranged from 0.049 and 0.054 (vs. TiLV index strain) to 0.159 (vs. TiLV/Maracas-2015-1 and -2, and FTGV/CobraB-2) (Table S3). Amino acid divergence ranged from 0.014 and 0.023 (vs. TiLV index strain) to 0.048 and 0.051 (vs. FTGV/Guppy/95/10/82) (Table S4). These findings reinforce the placement of AmnoonvirusEGY1F and -H in the genus *Tilapinevirus* as distinct variants within the genus. The amino acid-based phylogenetic tree (Figure 3) corroborates these findings, showing that AmnoonvirusEGY1F and -H form a distinct clade within the classified *Amnoonviridae* members. This distinct clustering at the protein level reinforces the genetic uniqueness of the Egyptian isolates and supports their classification within the *Tilapinevirus* genus, despite divergence from other known species within the genus.

**Figure 4:**
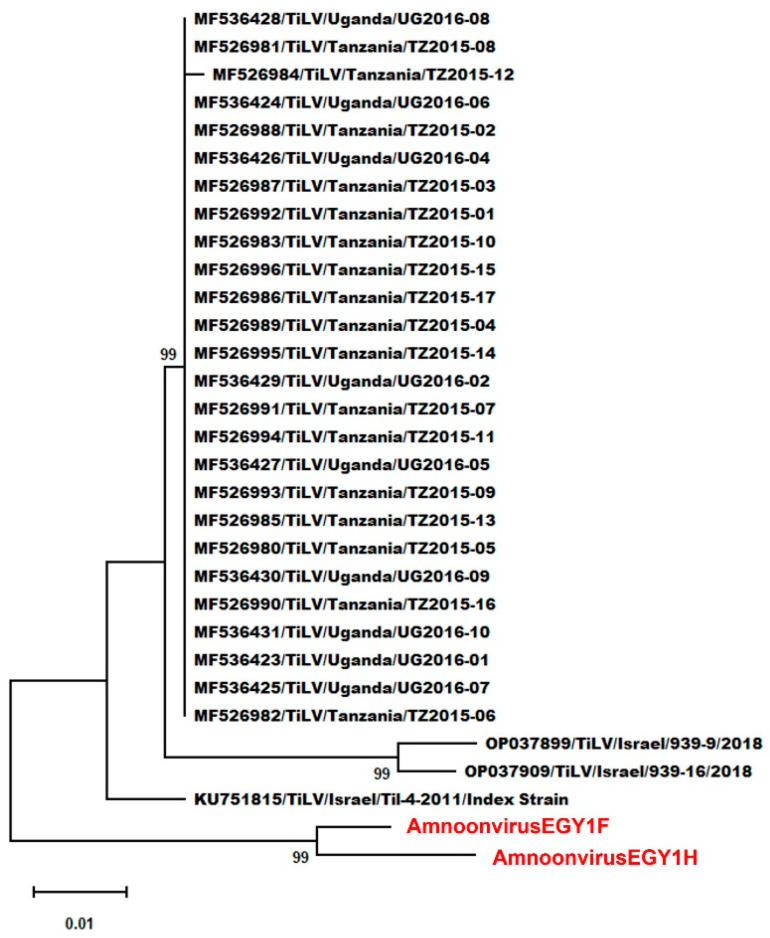
Maximum likelihood phylogram based on partial segment 2 nucleotide sequences, illustrating the phylogenetic relationships between the Egyptian amnoonviruses (AmnoonvirusEGY1F and -H, shown in red) and tilapia lake virus (TiLV) strains previously reported from Israel and Africa (Lake Victoria). Each viral isolate is labeled with its GenBank accession number, virus name, country of origin, and isolate designation. Branch lengths represent the number of nucleotide substitutions per site, and bootstrap support values ≥ 70% are shown at the nodes.

Finally, phylogenetic and genetic analyses based on partial segment 2 sequences from all TiLV isolates reported from Lake Victoria and representative Israeli TiLV isolates demonstrated that AmnoonvirusEGY1F and -H formed a well-supported, separate clade (Figure 4) from all viruses included in this analysis. Nucleotide sequence differences ranged from 0.057 and approximately 0.063 (vs. TiLV index strain and Lake Victoria isolates) to 0.071 and 0.079 (vs. TiLV/Israel/939-16/2018) (Table S5). Amino acid divergence ranged from 0.149 and 0.169 (vs. TiLV index strain) to 0.149 and 0.176 (vs. Lake Victoria isolates) to 0.184 and 0.203 (vs. TiLV/Israel/939-16/2018) (Table S6).

### 3.3. Predicting the 3-Dimensional Structure of PB2 Subunit of AmnoonEGY1F and -H

**Figure 5:**
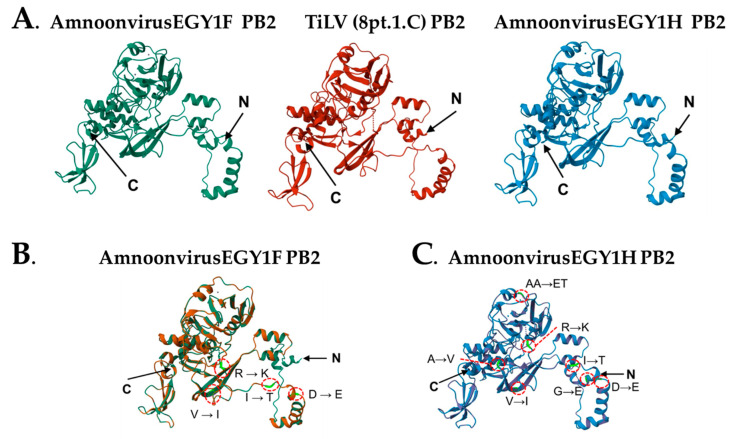
The 3D structures predicted by the SWISS-MODEL using the RNA-dependent RNA polymerase PB2 unit of the TiLV index strain as a reference template. (**A**) The 3D models of PB2 from AmnoonEGY1F (green), AmnoonEGY1H (blue), and TiLV index strain (maroon). N: The N-terminus or the first residue, C: the C-terminus or the last residue. (**B**) Predicted AmnoonvirusEGY1F structure showing the positions of some amino acid substitutions marked with dotted red circles. (**C**) Predicted AmnoonvirusEGY1H structure showing the positions of some amino acid substitutions marked with dotted red circles. Letters and small arrows refer to the amino acids substituted.

The protein sequence of AmnoonEGY1F PB2 spans nucleotides 55-1428 of the RNA sequence. Similarly, the protein sequence of AmnoonEGY1H PB2 spans the same nucleotide positions. As displayed in Figure 5A, the SWISS MODEL predicted the 3D structure of the PB2 proteins of both Egyptian strains and used the TiLV index strain as a reference for comparison. As in the case of TiLV, both AmnoonvirusEGY1F and -H PB2 subunits are composed of 457 amino acid residues and possess an N-terminal domain, two α-helical lid domains, and a number of C domains. The PB2 3D protein structure of both Egyptian strains exhibited the same topology of the TiLV PB2 predicted structure detailed in Arragain et al. [20]. The MolProbity index values validated the reliability of the three predicted PB2 protein structures, those being 1.11 in the case of AmnoonvirusEGY1F, 1.24 in the case of AmnoonvirusEGY1H, and 1.12 in the case of the TiLV index strain. A MolProbity score of ~1–2 is considered optimal, as it mimics the score obtained from a high-resolution crystal structure (http://proteiniq.io/app/molprobity, accessed on 7 December 2025). Despite the shared topology, there were some negligible differences among the three predicted structures. For example, the bond angles at positions C55–C57, C102–C103, C141–142, C317–C318, CC324–C325, and C331–C332 deviated significantly from ideal geometry in the TiLV index strain, but not in either AmnoonvirusEGY1 variant. On the contrary, two bond angles (C144 and C147) were deviated in both AmnoonvirusEGY1 variants but not in the TiLV index strain.

Comparative analysis of the PB2 protein sequences of AmnoonvirusEGY1H and -F relative to the TiLV index strain demonstrated that both strains shared six mutations (D55E, I61T, V117I, R140K, I228V, and R236K) (Figure 5B), while EGY1H had four additional substitutions (G5E, A122V, A230E, and A231T) (Figure 5C). The potential impacts of these mutations were predicted utilizing three bioinformatic tools, with each assessing one aspect: sequence-based tolerance assessment (SIFT), conservation-based assessment (ConSurf), and structure-based assessment (FoldX). SIFT analysis predicted that most shared mutations between the two variants are likely to be tolerated. However, the substitutions A230E and A231T in AmnoonvirusEGY1H were predicted to be less tolerated based on their physicochemical differences from the TiLV reference residues. Similarly, the ConSurf conservation analysis demonstrated that most mutated residues in both variants occur within regions of low to moderate evolutionary conservation, which is in line with their SIFT-predicted tolerance. On the contrary, mutated residues in positions 230 and 231 fell in a region of higher conservation, suggesting that these mutations can exert a significant impact compared to the other mutations shared between the two Egyptian variants (Figure S2). The potential negative impact on the AmnoonvirusEGY1H PB2 protein by A230E and A231T was further confirmed by the FoldX structure stability-based assessment. The A230E mutation added a negatively charged residue into a structurally constrained region. The A231T substitution introduced a polar side chain that could alter local hydrogen-bonding patterns. On the contrary, other shared mutations were either surface-exposed or within a side-chain region, and thus their disruptive effects on the protein structure were predicted to be minimal. Additionally, the FoldX analysis predicted structure energies for the TiLV index strain and AmnoonvirusEGY1F and -H to be 292.3, 282.89, and 277.86 kcal/mol, respectively.

### 3.4. Assessing the Predicted Differences in Antigenicity of the PB2 Subunit Between AmnoonvirusEGY1F and -H

The CTL and LBL epitopes on the PB2 proteins of the two Egyptian strains were compared to each other and to the TiLV index strain. Thirteen peptides in the TiLV index strain were identified as potential CTL epitopes, each consisting of nine amino acids. Of these, five exhibited varying degrees of antigenicity (Table 2). The same number of peptides were predicted from the PB2 sequences of both AmnoonvirusEGY1F and -H; however, a 14th CTL epitope of an identical sequence was identified in each of the two Egyptian strains but not in TiLV. This additional epitope in both cases was predicted to be non-antigenic. Further, the analysis predicted 29 potential LBL epitopes in the TiLV index strain and 28 in both amnoonvirus variants of this study, each comprising 16 amino acid residues. The additional LBL epitope unique to the index strain was predicted to be antigenic. Among the 28 shared epitopes, 23 were of identical sequence in all three strains and were classified as antigenic (Table 3). Additionally, several point mutations were observed within five of the predicted epitopes when AmnoonvirusEGY1F and -H were compared to one another or to the TiLV index strain. Although these variations did not change the overall antigenicity probabilities, they influenced the prediction confidence scores and the calculated probability values for protective antigenicity. Overall, 5 CTL and 14 LBL epitopes surpassed the antigenicity threshold of ≥0.4 in both AmnoonvirusEGY1 variants, with an additional antigenic LBL epitope which was unique to TiLV.

## 4. Discussion

This study focused on analyzing nucleotide and amino acid sequences of segment 2 which encodes the PB2 subunit of the heterotrimeric RNA-dependent-RNA polymerase (RdRp) of two amnoonvirus contigs originating from farmed Nile tilapia in Egypt. Segment 2 was chosen because its conserved sequence allows deciphering the evolutionary relationship between viruses within the order *Articulavirales* and also because its PB2-encoded protein is involved in various vital functions such as virus replication, pathogenicity, and evasion of host defense mechanisms [42]. In particular, the cap-snatching process that is orchestrated by PB2 is of paramount importance, as it enables the virus to gain capped primers to assemble its own messenger RNAs, synthesize its complementary RNA, and produce viral proteins [43,44]. Studies on other viruses within the order *Articulavirales* (e.g., influenza viruses) have shown that a change in specific amino acids in PB2 can increase the virus’s virulence and its ability to evade the host defense mechanisms [45]. It is for these reasons that this study is focused on the phylogenetic analysis of the full-length segment 2 and explores some proteomic characteristics of the encoded PB2 subunit protein of the recently detected amnoonvirus variants infecting Nile tilapia in Egypt.

As expected, the Illumina sequencing platform delivered high-throughput data of segment 2 sequences with remarkable accuracy. It was possible to assemble two contigs (AmnoonvirusEGY1F and -H) with each encompassing the full length of a segment 2 nucleotide sequence, attaining a Phred quality score of ≥Q30 (i.e., <0.1% error rate) and confirming the integrity of the assembly. Alignment of both contigs demonstrated that they are closely related variants (98%), probably of the same virus, yet there were multiple mutations that differentiate them from one another and from the TiLV index strain. For this reason, in further phylogenetic and genetic analyses performed in this study, each contig was analyzed independently.

Phylogenetic analysis of segment 2 sequences demonstrated that both AmnoonvirusEGY1F and -H are positioned within the *Amnoonviridae* family. Within the *Amnoonviridae*, both contigs formed a well-supported clade in the genus *Tilapinevirus*, the only ICTV-recognized genus in the family [7], along with TiLV and *T. poikilos*. Pairwise nucleotide distances further confirmed the divergence of both AmnoonvirusEGY1 variants of this study from both TiLV and *T. poikilos*. Additionally, both amnoonvirus variants of this study were divergent from all other unclassified amnoonviruses, proposed to be included in the family *Amnoonviridae* [10].

Further phylogenetic analyses that included members of the current (i.e., *T. tilapiae*) and proposed (i.e., *T. poikilos*) species within the genus *Tilapinevirus* showed AmnoonvirusEGY1F and -H forming a well-supported genetic divergence (in both nucleotide and amino acid sequences) that demarcated them from all other TiLV strains originating from several countries, whose segment 2 full sequences are available in public databases. It is noteworthy that TiLV strains from different geographic areas clustered into a number of well supported clades regardless of the region of their original detection, an indication of the presence of marked genetic heterogeneity among current TiLV strains. Similar genetic variations were also described by Chaput et al. [46] based on phylogenetic studies performed on a large number of TiLV strains. The authors attributed this genetic heterogeneity among TiLV isolates to a history of mutations and possibly recent reassortment because earlier studies did not show this level of genetic divergence in TiLV [13,47]. Carey et al. [48] suggested the observed variability among members of the order *Articulavirales* was due to the inability of RdRp to proofread the viral RNA, allowing mutations to happen, and predicted that these mutations may increase and could lead to the emergence of viral species with altered characteristics. Because genetic variants of TiLV may have altered characteristics such as virulence, host range, and immune evasion, it is imperative to determine which TiLV strains should continue to be listed as notifiable pathogens by the World Animal Health Organization and which of these genetic variants should be excluded from notification.

There was an obstacle in identifying the genetic relationship between the Egyptian strains and those detected and isolated from Lake Victoria in Uganda and Tanzania [15], as they share the Nile River basin. This is primarily due to the absence of complete genome sequences or even full segment sequences from the African strains. Therefore, we performed phylogenetic and genetic analyses based on partial segment 2 sequences, the only sequence available for the Lake Victoria TiLV strain, and included several strains from Israel. The analyses demonstrated that AmnoonvirusEGY1F and -H formed a well-supported, separate clade that demarcated them from the African and Israeli strains. Nucleotide and amino acid sequence differences suggest that the two Egyptian contigs, though divergent from all other viruses included in this study, exhibited a closer genetic relationship with the 26 Lake Victoria strains and the Israeli index strain than to the other two Israeli strains included (939-9/2018, 939-16/2018). These findings corroborate those of Chaput et al. [46] using partial segment 2 sequences, who reported that two African strains (from Tanzania and Uganda) clustered with one Israeli strain but not with other Israeli strains. Considering the close geographical proximity between Egypt and Israel, and the shared River Nile basin with Lake Victoria, it seems that there are ongoing genetic variations occurring among members of the genus *Tilapinevirus*, a matter that underscores the dire need for continued surveillance to determine the diversity, distribution, pathogenicity, and host range of amnoonviruses and their variants in order to limit their spread and threat to the tilapia aquaculture industry in Egypt and elsewhere.

Phylogenetic data generated in this study further confirm our previous findings [12] on the presence of an amnoonvirus in the Nile tilapia in Egypt that is divergent from all other amnoonviruses sequenced thus far. Further, the findings of this study also shed light on the increasing discovery of previously unknown amnoonviruses that have led to a number of proposals to the ICTV to recognize new genera (e.g., the *Lautavirus*, [8]) and species [9] in the family *Amnoonviridae.* In this same context, Petrone et al. [49] suggested the reclassification of a number of genera containing fish-pathogenic viruses (e.g., *Isavirus*, *Mykissvirus*, and *Sardinvirus*) currently placed in the family *Orthomyxoviridae* into the family *Amnoonviridae*, which was once believed to contain a single genus and a single species.

Despite their close genetic relatedness, the two Egyptian segment 2 contigs differed by 30 nucleotide polymorphisms (SNPs) when aligned. These substitutions were predominantly nonsynonymous, resulting in corresponding amino acid changes. The accumulation of mutations in RNA viruses is well documented, as their replication is inherently error-prone [50]. While certain mutations may negatively impact viral fitness by reducing replication efficiency, pathogenicity, or resistance to adverse environmental conditions [51], others can enhance capability by eliminating less-fit variants [52], promoting genetic diversity and evolutionary adaptation, and modifying viral antigenicity to facilitate evasion of the host immune response [50]. Studies on several orthomyxoviruses (e.g., influenza viruses [53] and thogotovirus [54]) demonstrated that mutations can lead to the emergence of variants with improved adaptation to surrounding environments which allow long-term survival of these strains [55]. Whether or not the genetic divergence of AmnoonvirusEGY1F and -H from all other known amnoonviruses is affecting their fitness, pathogenicity, or ability to evade the fish immune system remains to be determined.

Despite the documented genetic diversity among members of the genus *Tilapinevirus*, the effects and functional consequences of mutations on the fitness of amnoonviruses have not been studied. This is alarming because one of these viruses, TiLV, is notifiable by the World Animal Health Organization, and current diagnostic assays cannot differentiate between TiLV and other amnoonviruses that can be less pathogenic or even apathogenic. For this reason, we opted in this study to use reliable bioinformatic tools to predict if the amino acid substitution in the two Egyptian variants can alter the structure, function, or antigenicity of PB2-encoded proteins.

The SWISS-MODEL generated 3D protein structures with remarkable stereochemical accuracy, which is expected from this model. The predicted 3D structure of the PB2 proteins of both variants and that of TiLV as described by Arragain et al. [20] are almost identical. This is not unexpected because RdRp is conserved in viruses of the order *Articulavirales* that share functional and structural resemblances despite size differences [20]. While the differences in bond angle can be trivial in their effect on virus fitness, they further affirm the presence of subtle differences between the two variants and their divergence from the TiLV index strain.

In this study, we combined the prediction of three bioinformatic tools, thereby obtaining an integrated evaluation of each mutation effect, something that could not be achieved by using a single prediction tool. For example, SIFT is a strictly sequence-based approach that can use homologous sequences to predict potential function impact due to a substitution; however, it cannot predict the potential effect of a mutation on virus evolution or the protein structure stability. On the other hand, ConSurf can only predict evolutionary conservation profiling, while FoldX can qualitatively predict structural stability only. Therefore, a combined approach using multiple predictions is the optimal method to identify substitutions of concern and design future research accordingly. In this context, the presence of two serious substitutions in AmnoonvirusEGY1H (A230E and A231T) suggests that this strain experiences substantial functional and structural abnormalities. The A230E substitution in this strain introduced a negatively charged glutamate to replace a small, neutral alanine, which could alter local electrostatics, disrupt packing interactions, or interfere with nearby functional motifs. In the same context, the introduction of a polar hydroxyl group by A231T may affect hydrogen-bonding networks and local secondary structures. However, these predictions need to be followed by in vitro and in vivo experiments to confirm the in silico prediction. By the same token, most substitutions shared by both variants fell in moderately or poorly conserved regions, with their side-chain chemistry and conformation largely unchanged, as predicted by ConSurf. Despite the prediction of these substitutions as benign, it remains to be determined if PB2 folding, stability, or other catalytic functions are enhanced or diminished by mutations predicted to be tolerable. Therefore, the in silico analysis performed on PB2 proteins in this study provides a foundation for future experimental validation regarding both newly detected amnoonvirus variants from Egypt. In particular, the polymerase activity, viral replication, host range, and temperature sensitivity need to be tested to determine whether the PB2 mutations in EGY1H and EGY1F impacted the virus fitness. In this respect, Wu et al. [56] successfully applied a polymerase minigenome assay (i.e., PB2 function in isolation) to study PB2 mutations, polymerase activity in vitro, and host adaptation of another virus in the order *Articulavirales*, the influenza virus. This approach, along with reverse genetics and recombinant virus rescue methods [57,58,59,60], is currently being used in our laboratory to test if AmnoonvirusEGY1H is indeed impacted by A230E and A231T. The current study affirmed the divergence of AmnoonvirusEG1F and -H from TiLV and identified specific PB2 residues that merit targeted investigation.

The PB2 protein of the TiLV index strain and the study strains (AmnoonvirusEGY1F and -H) exhibited a largely similar repertoire of predicted CTL and LBL epitopes. However, several sequence variations were detected among the strains, leading to differences in prediction confidence scores and calculated probability values for protective antigenicity. Despite the fact that the variations noted between the three amnoonvirus strain appear to be minimal, they signify phenotypic changes in the encoded protein that may lead to altered immune response. While the immunoinformatic tools used in this study to identify CTL and LBL epitopes are originally designed for reverse vaccinology purposes and development of enhanced disease diagnostic reagents, they were employed in this study because their computational protocols rely not only on an epitope’s ability initiate an immune cascade [61], but also on the proteomic cleavage and transport efficiency of that protein, as well as its isoelectric point, hydrophobicity, and hydrophilicity [61], all of which are important phenotypic characteristics of a protein that affect its functions. Therefore, even minor changes in epitope prediction can shed light on potential changes in the protein structure and stereochemistry that determine the ability of fish to mount an effective immune response or enable the virus to evade the host’s defense mechanisms.

Of note, however, is the presence of point mutations in five of the LBL epitopes. LBL analysis showed one antigenic epitope identified in the TiLV index strain that was missing in the study isolates, suggesting that mutations in the study isolates may have reduced their overall antigenicity. How these mutations affect the humoral immune response of the host remains to be elucidated. It is believed that the antibody response is the major defense strategy by the host against TiLV [62,63,64], hence the importance of studying LBL epitopes and their role in immune evasion. In an earlier study, immunoinformatic tools were employed to predict CTL and LBL epitopes on TiLV-encoded PB1 proteins [65]. The authors reported the prediction of 47 CTL epitopes; 19 of them were antigenic, with the C scores of the top three ranging from 0.53 to 0.87 and prediction scores from 0.53 to 0.72. In contrast, predicted CTL epitopes on PB2 in this study were much fewer in number, albeit much higher in C scores (0.75 to 3.37), with the prediction scores ranging from 0.4 to 1.2. Differences were also observed in the LBL epitopes; 10 LBL epitopes were predicted on PB2 with an antigenicity prediction that ranged from 0.73 to 0.81, while the current study of encoded PB2 proteins showed 29 LBL predictions, with the antigenicity scores of the top three ranging from 0.8 to ~1.2. The findings of both studies point to variability in the antigenicity of proteins encoded by different RNA segments of amnoonviruses, an important finding that needs to be followed up for better understanding not only of virus–host interactions but also of amnoonvirus evolution and how accumulated mutations can affect virus fitness.

While this exploratory proteomic study verified the presence of differences between the two Egyptian virus variants and TiLV, it has also shed light on the emergence of novel amnoonviruses and the increased diversity in the family *Amnoonviridae.* Despite the fact that amnoonviruses have been detected in a large number of fish species and other lower vertebrates, we know little about their encoded protein functions and structure, a matter that is hampering the design of effective control strategies. The observed amino acid substitutions in the epitopes and elsewhere in this study serve as signals of rapid evolutionary change in amnoonviruses particularly those infecting tilapia spp., one of the most important fish species in global aquaculture.

## 5. Conclusions

The study provides detailed phylogenetic and genetic analyses of segment 2 from two variants of an amnoonvirus detected in farmed Nile tilapia in Egypt. Using both nucleotide and amino acid sequences, multiple phylogenetic trees were constructed, comparing the Egyptian strains to a wide range of amnoonviruses, including tilapia lake virus (TiLV) strains isolated from different geographic regions and fish species. In all trees constructed by both full-length and partial segment 2 sequences, the Egyptian strains clustered together, forming a distinct clade. Additional computational tools predicted the presence of variations between the two newly detected amnoonviruses and the TiLV index strain that included some mutations that may affect the function or structure of the PB2 encoded proteins. There is a dire need to determine the pathogenicity of both divergent amnoonviruses and their potential threat to tilapia aquaculture.

## Figures and Tables

**Table 2:** Predicted cytotoxic T lymphocyte (CTL) epitopes from the PB2 protein of the TiLV index strain and the two AmnoonvirusEGY1 variants of this study. Each 9-mer peptide is listed with its position, its C and prediction scores (the higher the score, the stronger the prediction). The antigenicity probability indicates the proportion of strains predicted to be antigenic. Ag: Antigenic. Nag: Non-antigenic.

Code	Sequence			Position	C-Score	Prediction Score	Antigenicity Probability
	TiLV-Index Strain	AmnoonvirusEGY1F	AmnoonvirusEGY1H				
CTL1	RTEVTITEY	RTEVTITEY	RTEVTITEY	11	3.3725	1.1884	Ag
CTL2	LISCSPGTY	LISCSPGTY	LISCSPGTY	417	1.9643	0.1875	NAg
CTL3	AIDLYNKGY	AIDLYNKGY	AIDLYNKGY	91	1.9333	1.0185	Ag
CTL4	TQEEAIDLY	TQEEAIDLY	TQEEAIDLY	87	1.9138	0.6925	Ag
CTL5	ALDRLRAAY	ALDRLRAAY	ALDRLRAAY	307	1.7074	0.1991	NAg
CTL6	YSSADNDKF	YSSADNDKF	YSSADNDKF	119	1.2992	−0.0697	NAg
CTL7	LSRSKWMGY	LSRSKWMGY	LSRSKWMGY	387	1.1921	0.9383	Ag
CTL8	KIGEFWHMY	KIGEFWHMY	KIGEFWHMY	178	1.1209	0.3330	NAg
CTL9	ITEYRSHTV	ITEYRSHTV	ITEYRSHTV	16	1.0411	0.4046	Ag
CTL10	LDDVVRGFY	LDDVVRGFY	LDDVVRGFY	370	0.9688	0.0196	NAg
CTL11	LTADKSLRK	LTADKSLRK	LTADKSLRK	32	0.9098	−0.1832	NAg
CTL12	YLDKAKVTV	YLDKAKVTV	YLDKAKVTV	378	0.8349	0.1002	NAg
CTL13	AFCDQGWVY	AFCDQGWVY	AFCDQGWVY	257	0.7526	0.2130	NAg
CTL14		RVVNEPIAY	RVVNEPIAY	111	0.8364	0.1433	NAg

**Table 3:** Comparison of predicted linear B-cell epitopes from the PB2 protein of the TiLV index strain, AmnoonvirusEGY1F, and -H strains. Amino-acid differences between isolates are highlighted in red. Prediction score and antigenic score are listed for each epitope (the higher the score, the stronger the prediction). The antigenicity probability indicates the proportion of strains predicted to be antigenic. Ag: Antigenic. NA: Non-antigenic.

Code	Sequence			Position	Prediction Score	Antigenic Score Prediction	Antigenicity Probability
	TiLV Index Strain	AmnoonvirusEGY1F	AmnoonvirusEGY1H				
LBL1	APTALDPFGGRAFCDQ	APTALDPFGGRAFCDQ	APTALDPFGGRAFCDQ	246	0.93	0.1850	NA
LBL2	LGPQGSANVSGSIHTA	LGPQGSANVSGSIHTA	LGPQGSANVSGSIHTA	292	0.91	0.3766	NA
LBL3	YRSHTVKDVHRSLLTA	YRSHTVKDVHRSLLTA	YRSHTVKDVHRSLLTA	19	0.88	0.1798	NA
LBL4	DRVVNEPVAYSSADND			110	0.89	0.0635	NA
		DRVVNEPIAYSSADND		110	0.86	0.1137	NA
			DRVVNEPIAYSSVDND	110	0.88	0.1314	NA
LBL5	KSKLRSQLSFRPGLTQ	KSKLRSQLSFRPGLTQ	KSKLRSQLSFRPGLTQ	73	0.87	1.1737	Ag
LBL6	KWMGYEDLPQKPPNGT	KWMGYEDLPQKPPNGT	KWMGYEDLPQKPPNGT	391	0.87	−0.0214	NA
LBL7	VGENFECDLDKRKLNI	VGENFECDLDKRKLNI	VGENFECDLDKRKLNI	336	0.87	0.5063	Ag
LBL8	DPSIAAAECPCRKVWE			225	0.87	0.1120	NA
		DPSVAAAECPCKKVWE		225	0.87	0.0044	NA
			DPSVAETECPCKKVWE	225	0.85	0.2171	NA
LBL9	QGWVYHRDVGYATANH	QGWVYHRDVGYATANH	QGWVYHRDVGYATANH	261	0.86	0.7384	Ag
LBL10	PGSFKDSLGFVIKIGE	PGSFKDSLGFVIKIGE	PGSFKDSLGFVIKIGE	166	0.85	0.6036	Ag
LBL11	ADRAFDTCESGFVRAI			139	0.86	−0.4642	NAg
		ADKAFDTCESGFVRAI	ADKAFDTCESGFVRAI	139	0.82	−0.5131	NAg
LBL12	TPCGFICCGPGSFKDS	TPCGFICCGPGSFKDS	TPCGFICCGPGSFKDS	157	0.85	0.4159	Ag
LBL13	VRAIPTTPCGFICCGP	VRAIPTTPCGFICCGP	VRAIPTTPCGFICCGP	151	0.85	0.0373	NAg
LBL14	EEAIDLYNKGYDGDSV	EEAIDLYNKGYDGDSV	EEAIDLYNKGYDGDSV	89	0.84	0.5497	Ag
LBL15	SGSIHTALDRLRAAYS	SGSIHTALDRLRAAYS	SGSIHTALDRLRAAYS	301	0.84	−0.0413	NAg
LBL16	GRTEVTITEYRSHTVK	GRTEVTITEYRSHTVK	GRTEVTITEYRSHTVK	10	0.84	0.5833	Ag
LBL17	NKGYDGDSVSGALQDR	NKGYDGDSVSGALQDR	NKGYDGDSVSGALQDR	96	0.83	0.8272	Ag
LBL18	HFVAVEDAKFLASKSP	HFVAVEDAKFLASKSP	HFVAVEDAKFLASKSP	191	0.83	0.5074	Ag
LBL19	SCSPGTYAKKRKVAVQ	SCSPGTYAKKRKVAVQ	SCSPGTYAKKRKVAVQ	419	0.82	0.8510	Ag
LBL20	GEFWHMYDGFQHFVAV	GEFWHMYDGFQHFVAV	GEFWHMYDGFQHFVAV	180	0.81	0.0023	NAg
LBL21	CRKVWEASFARAPTAL			235	0.81	−0.2495	NAg
		KKVWEASFARAPTALD	KKVWEASFARAPTALD	236	0.80	−0.0571	Nag
LBL22	DRFKDMRVENFREVAE	DRFKDMRVENFREVAE	DRFKDMRVENFREVAE	436	0.79	0.0715	NAg
LBL23	DKSLRKSFCFRNALNQ	DKSLRKSFCFRNALNQ	DKSLRKSFCFRNALNQ	35	0.79	0.2374	NAg
LBL24	AAYSRGTPASRSILQG	AAYSRGTPASRSILQG	AAYSRGTPASRSILQG	313	0.79	0.7658	Ag
LBL25	GFVIKIGEFWHMYDGF	GFVIKIGEFWHMYDGF	GFVIKIGEFWHMYDGF	174	0.76	0.4873	Ag
LBL26	QKPPNGTFYCRKRKAM	QKPPNGTFYCRKRKAM	QKPPNGTFYCRKRKAM	400	0.75	0.7505	Ag
LBL27	KLNIKALRSPERYITI	KLNIKALRSPERYITI	KLNIKALRSPERYITI	348	0.75	0.5070	Ag
LBL28	AYSSADNDKFHRGLAA	AYSSADNDKFHRGLAA		118	0.76	−0.1714	NAg
			AYSSVDNDKFHRGLAA	118	0.75	−0.1622	NAg
LBL29	LLPIRPKLESRVAVKK			58	0.84	1.1739	Ag

## Data Availability

The original contributions presented in this study are included in the article/Supplementary Materials, further inquiries can be directed to the corresponding author. Full length sequences of segment 2 of AmnoonvirusEGY1F and AmnoonvirusEGY1H are deposited at NCBI GenBank under the accession numbers PX458600 and PX458601, respectively.

## Supplementary Materials

The following supporting information can be downloaded at: https://www.mdpi.com/article/10.3390/microorganisms14020343/s1, Figure S1: Pairwise nucleotide alignment of the segment 2 contigs from AmnoonvirusEGY1F and -H using the NCBI Align Sequences (Nucleotide BLAST) tool. Dots represent identical nucleotides, and red letters indicate mismatched bases. The alignment shows 98% sequence identity with no gaps. Figure S2: ConSurf prediction of variable-conserved amino acids in the polymerase subunit 2 (PB2) domain. The prediction was performed with 35 homologous full-length PB2 sequences identified from the BLASTp results. The TiLV index strain, AmnoonvirusEGY1F and -H were also included. Table S1: Pairwise nucleotide sequence distances based on the segment 2 gene between the Egyptian amnoonvirus isolates (this study) and representative members of the order *Articulavirales*. Distances were calculated using the p-distance model. Lower values indicate higher sequence similarity. Table S2: Pairwise animo acid sequence distances based on the segment 2 gene between the Egyptian amnoonvirus isolates (this study) and representative members of the order *Articulavirales*. Distances were calculated using the p-distance model. Lower values indicate higher sequence similarity. Table S3: Pairwise nucleotide sequence distances between the Egyptian amnoonvirus isolates (this study) and different *Amnoonviridae* members, including TiLV, TiLV-like, and FTGV, with ISAV PB2 gene as outgroup. Distances were calculated using the p-distance model. Lower values indicate higher sequence similarity. Table S4: Pairwise amino acid sequence distances between the Egyptian amnoonvirus isolates (this study) and different *Amnoonviridae* members, including TiLV, TiLV-like, and FTGV, with ISAV as outgroup, by PB2 gene. Distances were calculated using the p-distance model. Lower values indicate higher sequence similarity. Table S5: Pairwise nucleotide sequence distances between the Egyptian amnoonvirus isolates (this study) and partial sequences of Israel and Africa (Lake of Victoria) isolates of segment 2. Distances were calculated using the p-distance model. Lower values indicate higher sequence similarity. Table S6: Pairwise amino acid sequence distances between the Egyptian amnoonvirus isolates (this study) and partial sequences of Israel and Africa (Lake of Victoria) isolates of segment 2. Distances were calculated using the p-distance model. Lower values indicate higher sequence similarity.
