# Supplementary material for: Phylogenetic and Proteomic Analyses of Segment 2 Sequence Reveals the Presence of Two Variants of a Divergent Amnoonvirus (Family: Amnoonviridae) Infecting Nile Tilapia (Oreochromis niloticus)
Source: Microorganisms. 2026 Feb 2;14(2):343. doi: 10.3390/microorganisms14020343 (PMC12943042; doi:10.3390/microorganisms14020343)
Supplement: Supplementary file 1 [file microorganisms-14-00343-s001.zip › microorganisms-4096421-supplementary.pdf]

Figure S1. Pairwise nucleotide alignment of the segment 2 contigs from AmnoonvirusEGY1F and -F strains using the NCBI Align Sequences (Nucleotide BLAST) tool. Dots represent identical nucleotides, and red letters indicate mismatched bases. The alignment shows 98% sequence identity with no gaps.

Score	Expect	Identities	Gaps	Strand
2551 bits(1381)	0.0	1441/1471(98%)	0/1471(0%)	Plus/Plus

```
EGY1F  1     CCAAATTTTACTCTCTATTACCAAATACATTTACTTCTGAAAATGAGTCAGTTTGGGAA  60
EGY1H  1     ....................................TC.............A....A...  60

EGY1F  61    ATCATTCAAGGGCAGAACTGAGGTCACAATAACCGAATATCGCTCTCATACTGTCAAAGA  120
EGY1H  61    G...........................................................  120

EGY1F  121   TGTGCACAGAAGCTTACTTACGGCTGACAAGTCTCTAAGGAAGTCATTTTGCTTTAGGAA  180
EGY1H  121   ............T...............................................  180

EGY1F  181   CGCCCTAAACCAGTTCTTGGATAAAGAGCTGCCCCTTTTGCCCACTCGGCCAAAGTTAGA  240
EGY1H  181   .................................T..........................  240

EGY1F  241   GTCCAGGGTTGCTGTGAAAAAGTCTAAGCTGAGAAGCCAGCTGTCGTTCAGACCCGGTTT  300
EGY1H  241   ...T..............G.................T.................T..C..  300

EGY1F  301   GACTCAAGAGGAAGCAATTGATCTTTACAACAAGGGCTATGATGGTGACAGCGTCTCAGG  360
EGY1H  301   ......G.....................................................  360

EGY1F  361   TGCCTTACAGGACAGGGTGGTCAACGAGCCTATAGCCTACTCAAGTGCAGATAATGACAA  420
EGY1H  361   .............................................C.T............  420

EGY1F  421   GTTTCACAGGGGCTTAGCAGCTCTAGGGTACACTTTAGCTGATAAAGCATTTGACACGTG  480
EGY1H  421   ............................................................  480

EGY1F  481   CGAATCCGGCTTCGTGAGAGCTATCCCTACTACTCCATGCGGGTTCATATGTTGTGGGCC  540
EGY1H  481   ..............................A.............................  540

EGY1F  541   AGGTTCTTTCAAAGATTCACTTGGATTTGTAATAAAAATTGGCGAATTCTGGCACATGTA  600
EGY1H  541   .......................................C....................  600

EGY1F  601   TGACGGGTTCCAACACTTCGTCGCTGTTGAGGACGCCAAGTTCCTGGCAAGTAAGTCTCC  660
EGY1H  601   ....................................T.......................  660

EGY1F  661   TTCGTTCTGGTTGGCAAAACGTCTTGCAAAGAGGCTGAATCTGGTCCCAAAGGAGGATCC  720
EGY1H  661   ............................................................  720

EGY1F  721   ATCTGTAGCAGCAGCTGAGTGCCCTTGTAAAAAAGTGTGGGAAGCTAGTTTTGCTAGAGC  780
EGY1H  721   ...........A.A..............................................  780

EGY1F  781   GCCTACTGCACTAGATCCATTTGGAGGCAGGGCCTTCTGCGACCAGGGGTGGGTGTACCA  840
EGY1H  781   ............G...............................................  840

EGY1F  841   CAGGGACGTAGGGTATGCAACTGCTAACCACATATCACAAGAAACACTTTTTCAACAGGC  900
EGY1H  841   .........G.............................G..............G..A..  900

EGY1F  901   ACTTTCAGTGAGGAACCTCGGACCACAGGGCAGTGCAAATGTCTCAGGTTCAATACATAC  960
EGY1H  901   G.......................G.....T...........T.................  960

EGY1F  961   AGCCCTGGACAGGCTCAGAGCAGCATACAGTAGGGGAACACCCGCCTCTAGATCTATACT  1020
EGY1H  961   ......................................................C.....  1020

EGY1F  1021  GCAAGGGCTTGCAAATCTCATCACACCTGTAGGTGAGAACTTTGAATGTGATCTCGACAA  1080
EGY1H  1021  ............................................................  1080

EGY1F  1081  AAGGAAGCTCAATATAAAGGCATTACGTTCTCCCGAGAGGTACATTACGATAGAGGGCCT  1140
EGY1H  1081  ............................................................  1140

EGY1F  1141  GGTTGTAAACTTGGACGATGTGGTTAGAGGGTTCTACCTTGACAAGGCGAAGGTCACTGT  1200
EGY1H  1141  ............................................................  1200

EGY1F  1201  TCTCTCGAGATCGAAGTGGATGGGTTACGAGGACCTGCCTCAGAAACCCCCGAATGGTAC  1260
EGY1H  1201  ............................................................  1260

EGY1F  1261  ATTTTACTGTAGAAAGAGGAAGGCAATGCTTCTCATCTCATGTAGTCCAGGCACGTACGC  1320
EGY1H  1261  ............................................................  1320

EGY1F  1321  AAAGAAGCGGAAAGTAGCAGTACAGGAGGATCGTTTTAAAGATATGAGGGTTGAGAACTT  1380
EGY1H  1321  ............................................................  1380

EGY1F  1381  CCGGGAAGTAGCGGAAAATATGGATCTAAATCAGTAGGGTTTCTTGGCAAAAGCCTTCAC  1440
EGY1H  1381  ............................................................  1440

EGY1F  1441  TATATATATGGTAATAATGAGAAAGATTTGC  1471
EGY1H  1441  ...............................  1471
```

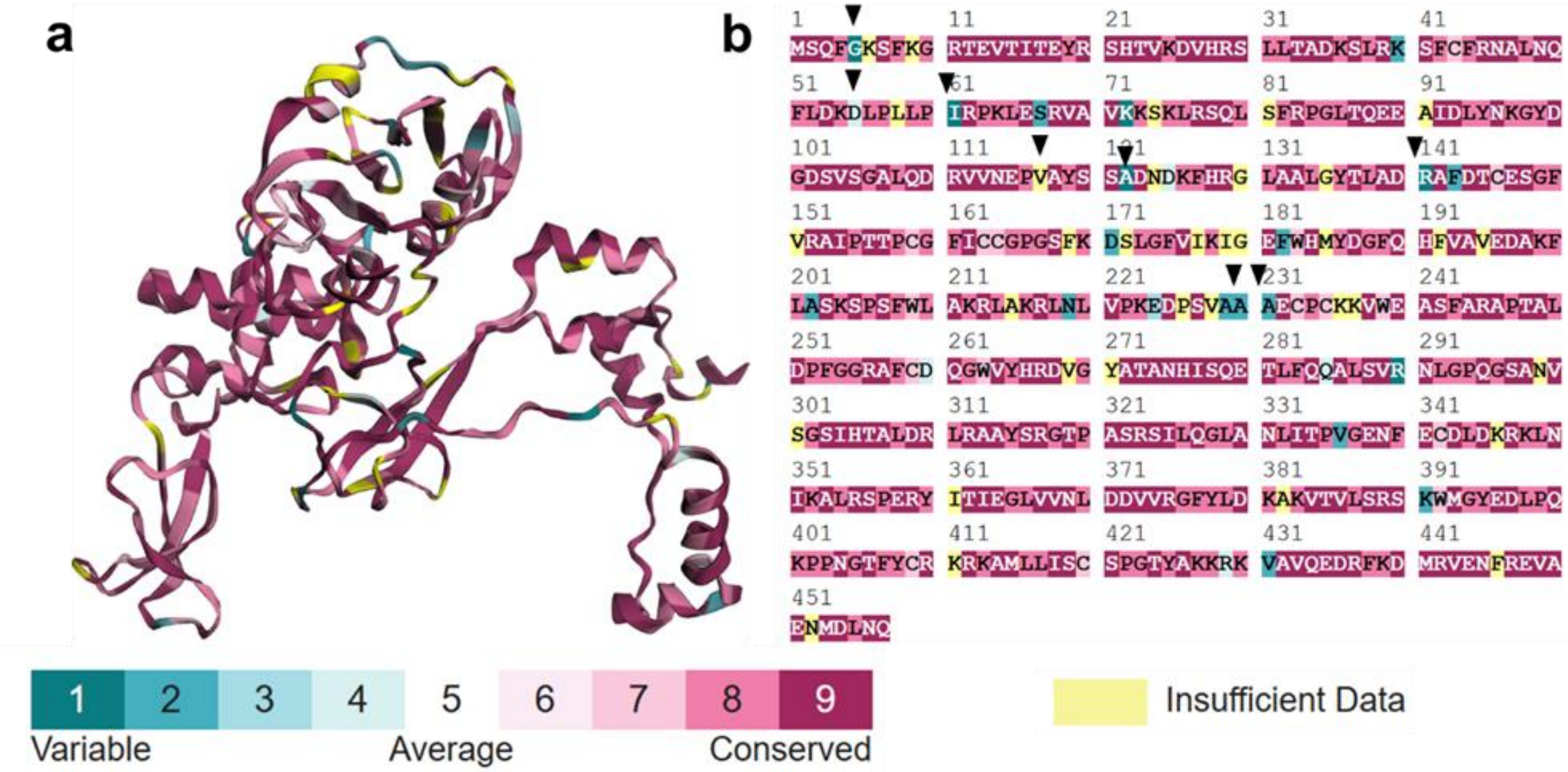

Figure S2. ConSurf prediction of variable-conserved amino acids in the polymerase subunit 2 (PB2) domain. The prediction was done with 35 homologous full-length PB2 sequences identified from the BLASTp results. TiLV index strain, AmnoonvirusEGY1F and -H were also included.

ble S1: Pairwise nucleotide sequence distances based on the segment 2 sequeence between the Egyptian Amnoonvirus isolates (this study) and representative members of the order *Articulavirales*. Distances were calculated using the p-distance model. Lower values indicate higher sequence similarity.

	AmnoonvirusEGY1F	AmnoonvirusEGY1H	TiLV/Index_Strain	TiLV_Like_Virus1	Influenza_A	Influenza_B	Influenza_C	Influenza_D	Rainbow_trout_orthomyxovirus1	Pilchard_orthomyxovirus	Infectious_salmon_anemia_virus	Stewartii_virus	Przewalskii_virus	Namensisvirus	Asotus_virus2	Asotus_virus1	Wenling_hagfish_influenza_virus	Wuhan_asiatic_toad_influenza_virus	Wuhan_spiny_eel_influenza_virus	Fancy_Tailed_Guppy_VirusB2
**AmnoonvirusEGY1F**																				
**AmnoonvirusEGY1H**	0.017																			
**TiLV/Index_Strain**	0.052	0.059																		
**TiLV_Like_Virus1**	0.175	0.181	0.172																	
**Influenza_A**	0.704	0.702	0.712	0.702																
**Influenza_B**	0.722	0.719	0.720	0.718	0.739															
**Influenza_C**	0.714	0.714	0.707	0.716	0.684	0.651														
**Influenza_D**	0.714	0.718	0.712	0.700	0.665	0.700	0.550													
**Rainbow_trout_orthomyxovirus1**	0.718	0.718	0.722	0.734	0.716	0.681	0.656	0.688												
**Pilchard_orthomyxovirus**	0.727	0.726	0.724	0.723	0.723	0.707	0.728	0.711	0.642											
**Infectious_salmon_anemia_virus**	0.738	0.742	0.735	0.737	0.699	0.718	0.714	0.734	0.669	0.691										
**Stewartii_virus**	0.715	0.716	0.707	0.702	0.754	0.739	0.749	0.733	0.746	0.685	0.741									
**Przewalskii_virus**	0.685	0.690	0.681	0.679	0.711	0.742	0.755	0.712	0.735	0.711	0.754	0.772								
**Namensisvirus**	0.651	0.648	0.656	0.636	0.742	0.691	0.727	0.734	0.723	0.735	0.738	0.728	0.677							
**Asotus_virus2**	0.672	0.676	0.664	0.659	0.685	0.753	0.727	0.726	0.754	0.730	0.749	0.714	0.629	0.731						
**Asotus_virus1**	0.680	0.681	0.681	0.687	0.730	0.754	0.731	0.749	0.739	0.698	0.777	0.737	0.595	0.703	0.573					
**Wenling_hagfish_influenza_virus**	0.681	0.683	0.683	0.692	0.681	0.700	0.684	0.685	0.734	0.754	0.741	0.753	0.735	0.735	0.723	0.726				
**Wuhan_asiatic_toad_influenza_virus**	0.719	0.715	0.718	0.698	0.690	0.673	0.625	0.668	0.679	0.712	0.720	0.757	0.750	0.710	0.708	0.742	0.691			
**Wuhan_spiny_eel_influenza_virus**	0.718	0.720	0.714	0.710	0.738	0.526	0.671	0.663	0.679	0.702	0.702	0.750	0.724	0.727	0.754	0.769	0.692	0.675		
**Fancy_Tailed_Guppy_VirusB2**	0.172	0.180	0.168	0.078	0.699	0.723	0.711	0.706	0.731	0.710	0.735	0.716	0.685	0.638	0.663	0.671	0.691	0.699	0.708	

Table S2: Pairwise animo acid sequence distances based on segment 2 sequence between the Egyptian Amnoonvirus isolates (this study) and representative members of the order *Articulavirales*. Distances were calculated using the p-distance model. Lower values indicate higher sequence similarity.

	AmnoonvirusEGY1F	AmnoonvirusEGY1H	TiLV/Index_Strain	TiLV_Like_Virus1	Influenza_A	Influenza_B	Influenza_C	Influenza_D	Rainbow_trout_orthomyxovirus1	Pilchard_orthomyxovirus	Infectious_salmon_anemia_virus	Stewartii_virus	Przewalskii_virus	Namensisvirus	Asotus_virus2	Asotus_virus1	Wenling_hagfish_influenza_virus	Wuhan_asiatic_toad_influenza_virus	Wuhan_spiny_eel_influenza_virus	Fancy_Tailed_Guppy_VirusB2
**AmnoonvirusEGY1F**																				
**AmnoonvirusEGY1H**	0.010																			
**TiLV/Index_Strain**	0.010	0.020																		
**TiLV_Like_Virus1**	0.088	0.088	0.078																	
**Influenza_A**	0.951	0.951	0.951	0.941																
**Influenza_B**	0.941	0.941	0.941	0.941	0.971															
**Influenza_C**	0.873	0.873	0.873	0.882	0.853	0.873														
**Influenza_D**	0.873	0.873	0.873	0.873	0.873	0.931	0.794													
**Rainbow_trout_orthomyxovirus1**	0.912	0.912	0.912	0.922	0.912	0.902	0.912	0.892												
**Pilchard_orthomyxovirus**	0.882	0.882	0.882	0.882	0.931	0.961	0.941	0.961	0.863											
**Infectious_salmon_anemia_virus**	0.912	0.912	0.912	0.931	0.912	0.931	0.922	0.931	0.882	0.902										
**Stewartii_virus**	0.912	0.912	0.912	0.902	0.902	0.961	0.902	0.882	0.971	0.922	0.941									
**Przewalskii_virus**	0.833	0.833	0.833	0.814	0.922	0.931	0.912	0.902	0.941	0.892	0.961	0.961								
**Namensisvirus**	0.784	0.775	0.784	0.775	0.951	0.912	0.912	0.873	0.931	0.902	0.951	0.882	0.882							
**Asotus_virus2**	0.853	0.853	0.853	0.843	0.853	0.941	0.892	0.873	0.922	0.902	0.951	0.873	0.775	0.882						
**Asotus_virus1**	0.873	0.873	0.873	0.882	0.902	0.941	0.961	0.922	0.931	0.902	0.980	0.951	0.657	0.922	0.676					
**Wenling_hagfish_influenza_virus**	0.804	0.814	0.804	0.843	0.902	0.873	0.892	0.902	0.902	0.961	0.902	0.941	0.922	0.951	0.882	0.882				
**Wuhan_asiatic_toad_influenza_virus**	0.912	0.912	0.912	0.912	0.912	0.902	0.833	0.892	0.873	0.922	0.931	0.951	0.941	0.951	0.931	0.922	0.892			
**Wuhan_spiny_eel_influenza_virus**	0.922	0.922	0.912	0.922	0.941	0.725	0.922	0.882	0.882	0.980	0.941	0.892	0.951	0.951	0.961	0.922	0.902	0.853		
**Fancy_Tailed_Guppy_VirusB2**	0.039	0.039	0.049	0.049	0.941	0.941	0.873	0.873	0.922	0.892	0.931	0.902	0.824	0.775	0.843	0.873	0.824	0.912	0.922	

Table S3: Pairwise nucleotide sequence distances between the Egyptian Amnoonvirus isolates (this study) and different Amnoonviridae members, including the TiLV, TiLV-like, and FTGV and ISAV as out group by PB2 gene. Distances were calculated using the p-distance model. Lower values indicate higher sequence similarity.

	AmnoonvirusEGY1F	AmnoonvirusEGY1H	KU751815/TiLV/Israel/Til-4-2011/Index_Strain	OQ437055/TiLV/Hong_Kong/TiLV-Israel-HK	MN939373/TiLV/Bangladesh/BD-2017	MZ297924/TiLV/India/IND-2018	ON376583/TiLV/Viet_Nam/RIA2-VN-2019	ON376573/TiLV/Viet_Nam/HB196-VN-2020	MK392373/TiLV/Ecuador/EC-2012	MT466438/TiLV/Bangladesh/BD-2017-181	MN687696/TiLV/Thailand/TH-2014	MT466448/TiLV/Bangladesh/BD-2019-E1	MN687706/TiLV/Thailand/TH-2015	MZ493942/TiLV/India/PMFGR:TL8	OP037899/TiLV/Israel/939-9/2018	PV356098/TiLV/Colombia/ICA2023	MN687686/TiLV/Thailand/TH-2013	MN687716/TiLV/Thailand/TH-2016-CU	MN687677/TiLV/Thailand/TH-2012	MK425011/TiLV/Peru/F3-4	KX631922/TiLV/Thailand/TV1	MN687756/TiLV/Thailand/TH-2018-k	MN687736/TiLV/Thailand/TH-2017	MN687726/TiLV/Thailand/TH-2016-CN	OP037909/TiLV/Israel/939-16/2018	MN687746/TiLV/Thailand/TH-2018-N	MT466458/TiLV/Bangladesh/BD-2019-E3	MH319379/TiLV/Thailand/WVL18053-01A	MN193524/TiLV/USA/WVL19054	MN193514/TiLV/USA/WVL19031-01A	MN687766/TiLV/Thailand/TH-2019	BK063211/TiLV/Trinidad_Tobago/Maracas-2015-2	BK063201/TiLV/Trinidad_Tobago/Maracas-2015-1	PP409986/FTGV/USA/Guppy/95/10/82	PP409996/FTGV/USA/CobraB-2	NC006505/ISAV/Norway/810/9/99
AmnoonvirusEGY1F																																				
AmnoonvirusEGY1H	0.021																																			
KU751815/TiLV/Israel/Til-4-2011/Index_Strain	0.049	0.054																																		
OQ437055/TiLV/Hong_Kong/TiLV-Israel-HK	0.051	0.053	0.001																																	
MN939373/TiLV/Bangladesh/BD-2017	0.058	0.057	0.026	0.025																																
MZ297924/TiLV/India/IND-2018	0.059	0.059	0.029	0.027	0.032																															
ON376583/TiLV/Viet_Nam/RIA2-VN-2019	0.070	0.071	0.052	0.051	0.055	0.057																														
ON376573/TiLV/Viet_Nam/HB196-VN-2020	0.075	0.078	0.056	0.056	0.062	0.063	0.007																													
MK392373/TiLV/Ecuador/EC-2012	0.055	0.056	0.021	0.019	0.026	0.030	0.055	0.061																												
MT466438/TiLV/Bangladesh/BD-2017-181	0.059	0.062	0.027	0.029	0.007	0.036	0.060	0.065	0.030																											
MN687696/TiLV/Thailand/TH-2014	0.059	0.065	0.023	0.025	0.028	0.031	0.056	0.061	0.031	0.029																										
MT466448/TiLV/Bangladesh/BD-2019-E1	0.059	0.063	0.029	0.031	0.010	0.038	0.062	0.067	0.032	0.010	0.032																									
MN687706/TiLV/Thailand/TH-2015	0.059	0.064	0.028	0.029	0.031	0.032	0.061	0.065	0.032	0.032	0.028	0.035																								
MZ493942/TiLV/India/PMFGR:TL8	0.059	0.063	0.029	0.029	0.032	0.022	0.058	0.062	0.032	0.032	0.031	0.035	0.025																							
OP037899/TiLV/Israel/939-9/2018	0.062	0.067	0.034	0.033	0.037	0.043	0.068	0.074	0.042	0.041	0.042	0.043	0.040	0.045																						
PV356098/TiLV/Colombia/ICA2023	0.076	0.076	0.047	0.045	0.051	0.055	0.070	0.075	0.034	0.054	0.054	0.054	0.054	0.054	0.061																					
MN687686/TiLV/Thailand/TH-2013	0.061	0.066	0.025	0.026	0.029	0.032	0.058	0.062	0.032	0.030	0.001	0.034	0.029	0.032	0.043	0.056																				
MN687716/TiLV/Thailand/TH-2016-CU	0.061	0.065	0.032	0.033	0.035	0.037	0.062	0.066	0.038	0.036	0.034	0.040	0.026	0.034	0.048	0.056	0.035																			
MN687677/TiLV/Thailand/TH-2012	0.062	0.067	0.026	0.027	0.030	0.033	0.059	0.063	0.032	0.031	0.002	0.034	0.030	0.033	0.044	0.056	0.004	0.036																		
MK425011/TiLV/Peru/F3-4	0.063	0.065	0.034	0.035	0.043	0.043	0.065	0.070	0.022	0.043	0.043	0.045	0.042	0.045	0.054	0.034	0.044	0.043	0.043																	
KX631922/TiLV/Thailand/TV1	0.062	0.063	0.034	0.034	0.037	0.039	0.067	0.072	0.041	0.040	0.037	0.043	0.029	0.036	0.044	0.057	0.039	0.031	0.040	0.045																
MN687756/TiLV/Thailand/TH-2018-k	0.064	0.065	0.031	0.032	0.041	0.040	0.062	0.065	0.032	0.042	0.040	0.044	0.041	0.041	0.052	0.052	0.041	0.044	0.042	0.043	0.051															
MN687736/TiLV/Thailand/TH-2017	0.064	0.068	0.034	0.035	0.037	0.036	0.064	0.068	0.038	0.037	0.031	0.041	0.012	0.025	0.045	0.059	0.032	0.029	0.033	0.046	0.032	0.047														
MN687726/TiLV/Thailand/TH-2016-CN	0.066	0.072	0.034	0.036	0.038	0.043	0.062	0.067	0.038	0.039	0.037	0.043	0.034	0.043	0.052	0.059	0.038	0.038	0.039	0.047	0.048	0.045	0.040													
OP037909/TiLV/Israel/939-16/2018	0.067	0.071	0.042	0.040	0.043	0.048	0.069	0.076	0.048	0.048	0.048	0.049	0.043	0.046	0.021	0.061	0.048	0.050	0.050	0.056	0.050	0.058	0.048	0.052												
MN687746/TiLV/Thailand/TH-2018-N	0.068	0.074	0.039	0.040	0.040	0.046	0.066	0.070	0.043	0.041	0.039	0.045	0.038	0.042	0.051	0.058	0.040	0.039	0.041	0.049	0.048	0.046	0.043	0.032	0.053											
MT466458/TiLV/Bangladesh/BD-2019-E3	0.069	0.073	0.037	0.039	0.018	0.046	0.065	0.070	0.039	0.018	0.040	0.014	0.042	0.042	0.051	0.056	0.042	0.048	0.041	0.051	0.051	0.049	0.048	0.048	0.056	0.051										
MH319379/TiLV/Thailand/WVL18053-01A	0.070	0.070	0.040	0.038	0.037	0.043	0.065	0.073	0.045	0.041	0.043	0.045	0.036	0.040	0.053	0.060	0.045	0.014	0.045	0.054	0.040	0.052	0.039	0.048	0.054	0.048	0.053									
MN193524/TiLV/USA/WVL19054	0.070	0.073	0.040	0.039	0.037	0.043	0.065	0.070	0.042	0.041	0.040	0.045	0.040	0.042	0.050	0.058	0.042	0.040	0.043	0.052	0.048	0.048	0.045	0.033	0.051	0.007	0.051	0.047								
MN193514/TiLV/USA/WVL19031-01A	0.071	0.074	0.043	0.041	0.041	0.047	0.065	0.071	0.044	0.045	0.041	0.048	0.042	0.044	0.051	0.060	0.043	0.043	0.043	0.054	0.051	0.050	0.047	0.035	0.052	0.010	0.055	0.048	0.010							
MN687766/TiLV/Thailand/TH-2019	0.073	0.078	0.044	0.045	0.045	0.051	0.070	0.074	0.048	0.046	0.040	0.050	0.043	0.047	0.055	0.063	0.041	0.044	0.042	0.054	0.054	0.051	0.048	0.035	0.055	0.011	0.055	0.051	0.014	0.009						
BK063211/TiLV/Trinidad_Tobago/Maracas-2015-2	0.159	0.159	0.153	0.152	0.148	0.147	0.155	0.158	0.154	0.152	0.156	0.152	0.155	0.152	0.152	0.166	0.158	0.158	0.157	0.159	0.153	0.159	0.155	0.163	0.158	0.161	0.158	0.162	0.161	0.158	0.163					
BK063201/TiLV/Trinidad_Tobago/Maracas-2015-1	0.159	0.159	0.153	0.152	0.148	0.147	0.155	0.158	0.154	0.152	0.156	0.152	0.155	0.152	0.152	0.166	0.158	0.158	0.157	0.159	0.153	0.159	0.155	0.163	0.158	0.161	0.158	0.162	0.161	0.158	0.163	0.001				
PP409986/FTGV/USA/Guppy/95/10/82	0.153	0.155	0.150	0.149	0.150	0.149	0.150	0.155	0.150	0.154	0.152	0.154	0.152	0.147	0.160	0.158	0.152	0.152	0.152	0.155	0.150	0.152	0.151	0.157	0.161	0.155	0.159	0.158	0.155	0.151	0.156	0.055	0.055			

PP409996/FTGV/USA/CobraB-2	0.159	0.159	0.151	0.150	0.152	0.150	0.155	0.160	0.155	0.156	0.152	0.156	0.152	0.150	0.161	0.163	0.154	0.153	0.155	0.165	0.150	0.152	0.153	0.161	0.159	0.158	0.159	0.156	0.158	0.153	0.157	0.065	0.065	0.063	
NC006505/ISAV/Norway/810/9/99	0.547	0.546	0.535	0.534	0.539	0.546	0.549	0.549	0.537	0.540	0.539	0.542	0.545	0.545	0.549	0.539	0.540	0.548	0.540	0.538	0.542	0.548	0.547	0.548	0.549	0.548	0.545	0.548	0.548	0.548	0.548	0.555	0.555	0.548	0.559

Table S4: Pairwise amino acid sequence distances between the Egyptian Amnoonvirus isolates (this study) and different Amnoonviridae members, including the TiLV, TiLV-like, and FTGV and ISAV as out group by PB2 gene. Distances were calculated using the p-distance model. Lower values indicate higher sequence similarity.

	AmnoonvirusEGY1F	AmnoonvirusEGY1H	KU751815/TiLV/Israel/Til-4-2011/Index_Strain	OQ437055/TiLV/Hong_Kong/TiLV-Israel-HK	MN939373/TiLV/Bangladesh/BD-2017	MZ297924/TiLV/India/IND-2018	ON376583/TiLV/Viet_Nam/RIA2-VN-2019	ON376573/TiLV/Viet_Nam/HB196-VN-2020	MK392373/TiLV/Ecuador/EC-2012	MT466438/TiLV/Bangladesh/BD-2017-181	MN687696/TiLV/Thailand/TH-2014	MT466448/TiLV/Bangladesh/BD-2019-E1	MN687706/TiLV/Thailand/TH-2015	MZ493942/TiLV/India/PMFGR:TL8	OP037899/TiLV/Israel/939-9/2018	PV356098/TiLV/Colombia/ICA2023	MN687686/TiLV/Thailand/TH-2013	MN687716/TiLV/Thailand/TH-2016-CU	MN687677/TiLV/Thailand/TH-2012	MK425011/TiLV/Peru/F3-4	KX631922/TiLV/Thailand/TV1	MN687756/TiLV/Thailand/TH-2018-k	MN687736/TiLV/Thailand/TH-2017	MN687726/TiLV/Thailand/TH-2016-CN	OP037909/TiLV/Israel/939-16/2018	MN687746/TiLV/Thailand/TH-2018-N	MT466458/TiLV/Bangladesh/BD-2019-E3	MH319379/TiLV/Thailand/WVL18053-01A	MN193524/TiLV/USA/WVL19054	MN193514/TiLV/USA/WVL19031-01A	MN687766/TiLV/Thailand/TH-2019	BK063211/TiLV/Trinidad_Tobago/Maracas-2015-2	BK063201/TiLV/Trinidad_Tobago/Maracas-2015-1	PP409986/FTGV/USA/Guppy/95/10/82	PP409996/FTGV/USA/CobraB-2	NC006505/ISAV/Norway/810/9/99
AmnoonvirusEGY1F																																			
AmnoonvirusEGY1H	0.009																																		
KU751815/TiLV/Israel/Til-4-2011/Index_Strain	0.014	0.023																																	
OQ437055/TiLV/Hong_Kong/TiLV-Israel-HK	0.016	0.021	0.002																																
MN939373/TiLV/Bangladesh/BD-2017	0.009	0.014	0.009	0.007																															
MZ297924/TiLV/India/IND-2018	0.011	0.016	0.011	0.009	0.007																														
ON376583/TiLV/Viet_Nam/RIA2-VN-2019	0.018	0.023	0.018	0.016	0.009	0.011																													
ON376573/TiLV/Viet_Nam/HB196-VN-2020	0.028	0.032	0.028	0.025	0.018	0.021	0.009																												
MK392373/TiLV/Ecuador/EC-2012	0.009	0.014	0.009	0.007	0.000	0.007	0.009	0.018																											
MT466438/TiLV/Bangladesh/BD-2017-181	0.011	0.021	0.011	0.014	0.007	0.014	0.016	0.025	0.007																										
MN687696/TiLV/Thailand/TH-2014	0.014	0.023	0.009	0.011	0.009	0.007	0.014	0.023	0.009	0.011																									
MT466448/TiLV/Bangladesh/BD-2019-E1	0.009	0.018	0.009	0.011	0.005	0.011	0.014	0.023	0.005	0.007	0.009																								
MN687706/TiLV/Thailand/TH-2015	0.016	0.025	0.011	0.014	0.011	0.014	0.016	0.025	0.011	0.014	0.011	0.011																							
MZ493942/TiLV/India/PMFGR:TL8	0.016	0.021	0.011	0.009	0.007	0.005	0.011	0.021	0.007	0.014	0.007	0.011	0.014																						
OP037899/TiLV/Israel/939-9/2018	0.014	0.018	0.009	0.007	0.005	0.007	0.014	0.023	0.005	0.011	0.009	0.009	0.011	0.007																					
PV356098/TiLV/Colombia/ICA2023	0.021	0.025	0.016	0.014	0.011	0.014	0.021	0.030	0.011	0.018	0.016	0.016	0.018	0.014	0.011																				
MN687686/TiLV/Thailand/TH-2013	0.016	0.025	0.011	0.014	0.011	0.009	0.016	0.025	0.011	0.014	0.002	0.011	0.014	0.009	0.011	0.018																			
MN687716/TiLV/Thailand/TH-2016-CU	0.011	0.021	0.007	0.009	0.007	0.009	0.016	0.025	0.007	0.009	0.007	0.007	0.009	0.009	0.007	0.014	0.009																		
MN687677/TiLV/Thailand/TH-2012	0.018	0.028	0.014	0.016	0.014	0.011	0.018	0.028	0.014	0.016	0.005	0.014	0.016	0.011	0.014	0.021	0.007	0.011																	
MK425011/TiLV/Peru/F3-4	0.018	0.028	0.014	0.016	0.014	0.016	0.018	0.028	0.014	0.016	0.014	0.014	0.011	0.016	0.014	0.016	0.016	0.011	0.018																
KX631922/TiLV/Thailand/TV1	0.016	0.016	0.011	0.009	0.007	0.009	0.016	0.025	0.007	0.014	0.011	0.011	0.014	0.009	0.007	0.014	0.014	0.009	0.016	0.016															
MN687756/TiLV/Thailand/TH-2018-k	0.014	0.023	0.009	0.011	0.009	0.011	0.018	0.023	0.009	0.011	0.009	0.009	0.011	0.011	0.009	0.016	0.011	0.007	0.014	0.014	0.011														
MN687736/TiLV/Thailand/TH-2017	0.016	0.025	0.011	0.014	0.011	0.009	0.011	0.021	0.011	0.014	0.007	0.011	0.009	0.005	0.011	0.018	0.009	0.009	0.011	0.011	0.014	0.011													
MN687726/TiLV/Thailand/TH-2016-CN	0.021	0.030	0.016	0.018	0.016	0.018	0.021	0.030	0.016	0.018	0.016	0.016	0.014	0.018	0.016	0.023	0.018	0.014	0.021	0.016	0.018	0.016	0.014												
OP037909/TiLV/Israel/939-16/2018	0.012	0.016	0.007	0.005	0.002	0.005	0.012	0.021	0.002	0.009	0.007	0.007	0.009	0.005	0.002	0.009	0.009	0.005	0.012	0.012	0.005	0.007	0.009	0.014											
MN687746/TiLV/Thailand/TH-2018-N	0.014	0.023	0.009	0.011	0.009	0.011	0.014	0.023	0.009	0.011	0.009	0.009	0.007	0.011	0.009	0.016	0.011	0.007	0.014	0.009	0.011	0.009	0.007	0.011	0.007										
MT466458/TiLV/Bangladesh/BD-2019-E3	0.011	0.021	0.011	0.014	0.007	0.014	0.014	0.023	0.007	0.009	0.011	0.007	0.011	0.014	0.011	0.018	0.014	0.009	0.011	0.014	0.014	0.011	0.011	0.016	0.009	0.009									
MH319379/TiLV/Thailand/WVL18053-01A	0.018	0.023	0.009	0.007	0.009	0.011	0.018	0.028	0.009	0.016	0.014	0.014	0.016	0.011	0.009	0.016	0.016	0.007	0.018	0.018	0.011	0.014	0.016	0.018	0.007	0.014	0.016								
MN193524/TiLV/USA/WVL19054	0.016	0.018	0.011	0.009	0.007	0.009	0.011	0.021	0.007	0.014	0.011	0.011	0.009	0.009	0.007	0.014	0.014	0.009	0.016	0.011	0.009	0.011	0.009	0.014	0.005	0.007	0.011	0.011							
MN193514/TiLV/USA/WVL19031-01A	0.016	0.021	0.011	0.009	0.007	0.009	0.011	0.021	0.007	0.014	0.011	0.011	0.009	0.009	0.007	0.014	0.014	0.009	0.016	0.011	0.009	0.011	0.009	0.014	0.005	0.007	0.011	0.011	0.005						
MN687766/TiLV/Thailand/TH-2019	0.014	0.023	0.009	0.011	0.009	0.011	0.014	0.023	0.009	0.011	0.009	0.009	0.007	0.011	0.009	0.016	0.011	0.007	0.014	0.009	0.011	0.009	0.007	0.011	0.007	0.005	0.009	0.014	0.007	0.007					
BK063211/TiLV/Trinidad_Tobago/Maracas-2015-2	0.044	0.046	0.039	0.037	0.034	0.034	0.034	0.039	0.034	0.041	0.037	0.039	0.041	0.034	0.034	0.037	0.039	0.037	0.041	0.044	0.037	0.039	0.039	0.044	0.032	0.039	0.041	0.039	0.037	0.037	0.039				
BK063201/TiLV/Trinidad_Tobago/Maracas-2015-1	0.044	0.046	0.039	0.037	0.034	0.034	0.034	0.039	0.034	0.041	0.037	0.039	0.041	0.034	0.034	0.037	0.039	0.037	0.041	0.044	0.037	0.039	0.039	0.044	0.032	0.039	0.041	0.039	0.037	0.037	0.039	0.005			
PP409986/FTGV/USA/Guppy/95/10/82	0.048	0.051	0.048	0.046	0.044	0.039	0.041	0.046	0.044	0.051	0.046	0.048	0.048	0.044	0.044	0.046	0.048	0.046	0.051	0.051	0.046	0.048	0.046	0.051	0.041	0.046	0.048	0.048	0.044	0.044	0.046	0.023	0.023		
PP409996/FTGV/USA/CobraB-2	0.041	0.039	0.041	0.039	0.032	0.037	0.032	0.037	0.032	0.039	0.039	0.037	0.041	0.037	0.037	0.037	0.041	0.039	0.044	0.044	0.039	0.041	0.039	0.046	0.035	0.039	0.037	0.041	0.034	0.037	0.039	0.023	0.023	0.025	
NC006505/ISAV/Norway/810/9/99	0.788	0.788	0.790	0.790	0.788	0.788	0.785	0.785	0.788	0.785	0.788	0.790	0.783	0.788	0.788	0.790	0.788	0.790	0.790	0.790	0.785	0.788	0.785	0.785	0.787	0.785	0.790	0.792	0.785	0.785	0.783	0.790	0.790	0.792	0.785

Table S5: Pairwise nucleotide sequence distances between the Egyptian Amnoonvirus isolates (this study) and partial sequences of Israel and Africa (Lake of Victoria) isolates by PB2 gene. Distances were calculated using the p-distance model. Lower values indicate higher sequence similarity.

	AmnoonvirusEGY1F	AmnoonvirusEGY1H	KU751815/TiLV/Israel/Til-4-2011/Index_Strain	OP037899/TiLV/Israel/939-9/2018	OP037909/TiLV/Israel/939-16/2018	MF536423/TiLV/Uganda/UG2016-01	MF536424/TiLV/Uganda/UG2016-06	MF536425/TiLV/Uganda/UG2016-07	MF536426/TiLV/Uganda/UG2016-04	MF536427/TiLV/Uganda/UG2016-05	MF536428/TiLV/Uganda/UG2016-08	MF536429/TiLV/Uganda/UG2016-02	MF536430/TiLV/Uganda/UG2016-09	MF536431/TiLV/Uganda/UG2016-10	MF526980/TiLV/Tanzania/TZ2015-05	MF526981/TiLV/Tanzania/TZ2015-08	MF526982/TiLV/Tanzania/TZ2015-06	MF526983/TiLV/Tanzania/TZ2015-10	MF526984/TiLV/Tanzania/TZ2015-12	MF526985/TiLV/Tanzania/TZ2015-13	MF526986/TiLV/Tanzania/TZ2015-17	MF526987/TiLV/Tanzania/TZ2015-03	MF526988/TiLV/Tanzania/TZ2015-02	MF526989/TiLV/Tanzania/TZ2015-04	MF526990/TiLV/Tanzania/TZ2015-16	MF526991/TiLV/Tanzania/TZ2015-07	MF526992/TiLV/Tanzania/TZ2015-01	MF526993/TiLV/Tanzania/TZ2015-09	MF526994/TiLV/Tanzania/TZ2015-11	MF526995/TiLV/Tanzania/TZ2015-14	MF526996/TiLV/Tanzania/TZ2015-15
**AmnoonvirusEGY1F**																															
**AmnoonvirusEGY1H**	0.024																														
**KU751815/TiLV/Israel/Til-4-2011/Index_Strain**	0.057	0.063																													
**OP037899/TiLV/Israel/939-9/2018**	0.069	0.077	0.043																												
**OP037909/TiLV/Israel/939-16/2018**	0.071	0.079	0.045	0.014																											
**MF536423/TiLV/Uganda/UG2016-01**	0.057	0.065	0.016	0.035	0.033																										
**MF536424/TiLV/Uganda/UG2016-06**	0.057	0.065	0.016	0.035	0.033	0.000																									
**MF536425/TiLV/Uganda/UG2016-07**	0.057	0.065	0.016	0.035	0.033	0.000	0.000																								
**MF536426/TiLV/Uganda/UG2016-04**	0.057	0.065	0.016	0.035	0.033	0.000	0.000	0.000																							
**MF536427/TiLV/Uganda/UG2016-05**	0.057	0.065	0.016	0.035	0.033	0.000	0.000	0.000	0.000																						
**MF536428/TiLV/Uganda/UG2016-08**	0.057	0.065	0.016	0.035	0.033	0.000	0.000	0.000	0.000	0.000																					
**MF536429/TiLV/Uganda/UG2016-02**	0.057	0.065	0.016	0.035	0.033	0.000	0.000	0.000	0.000	0.000	0.000																				
**MF536430/TiLV/Uganda/UG2016-09**	0.057	0.065	0.016	0.035	0.033	0.000	0.000	0.000	0.000	0.000	0.000	0.000																			
**MF536431/TiLV/Uganda/UG2016-10**	0.057	0.065	0.016	0.035	0.033	0.000	0.000	0.000	0.000	0.000	0.000	0.000	0.000																		
**MF526980/TiLV/Tanzania/TZ2015-05**	0.057	0.065	0.016	0.035	0.033	0.000	0.000	0.000	0.000	0.000	0.000	0.000	0.000	0.000																	
**MF526981/TiLV/Tanzania/TZ2015-08**	0.057	0.065	0.016	0.035	0.033	0.000	0.000	0.000	0.000	0.000	0.000	0.000	0.000	0.000	0.000																
**MF526982/TiLV/Tanzania/TZ2015-06**	0.057	0.065	0.016	0.035	0.033	0.000	0.000	0.000	0.000	0.000	0.000	0.000	0.000	0.000	0.000	0.000															
**MF526983/TiLV/Tanzania/TZ2015-10**	0.057	0.065	0.016	0.035	0.033	0.000	0.000	0.000	0.000	0.000	0.000	0.000	0.000	0.000	0.000	0.000	0.000														
**MF526984/TiLV/Tanzania/TZ2015-12**	0.059	0.067	0.018	0.037	0.035	0.002	0.002	0.002	0.002	0.002	0.002	0.002	0.002	0.002	0.002	0.002	0.002	0.002													
**MF526985/TiLV/Tanzania/TZ2015-13**	0.057	0.065	0.016	0.035	0.033	0.000	0.000	0.000	0.000	0.000	0.000	0.000	0.000	0.000	0.000	0.000	0.000	0.000	0.002												
**MF526986/TiLV/Tanzania/TZ2015-17**	0.057	0.065	0.016	0.035	0.033	0.000	0.000	0.000	0.000	0.000	0.000	0.000	0.000	0.000	0.000	0.000	0.000	0.000	0.002	0.000											
**MF526987/TiLV/Tanzania/TZ2015-03**	0.057	0.065	0.016	0.035	0.033	0.000	0.000	0.000	0.000	0.000	0.000	0.000	0.000	0.000	0.000	0.000	0.000	0.000	0.002	0.000	0.000										
**MF526988/TiLV/Tanzania/TZ2015-02**	0.057	0.065	0.016	0.035	0.033	0.000	0.000	0.000	0.000	0.000	0.000	0.000	0.000	0.000	0.000	0.000	0.000	0.000	0.002	0.000	0.000	0.000									
**MF526989/TiLV/Tanzania/TZ2015-04**	0.057	0.065	0.016	0.035	0.033	0.000	0.000	0.000	0.000	0.000	0.000	0.000	0.000	0.000	0.000	0.000	0.000	0.000	0.002	0.000	0.000	0.000	0.000								
**MF526990/TiLV/Tanzania/TZ2015-16**	0.057	0.065	0.016	0.035	0.033	0.000	0.000	0.000	0.000	0.000	0.000	0.000	0.000	0.000	0.000	0.000	0.000	0.000	0.002	0.000	0.000	0.000	0.000	0.000							
**MF526991/TiLV/Tanzania/TZ2015-07**	0.057	0.065	0.016	0.035	0.033	0.000	0.000	0.000	0.000	0.000	0.000	0.000	0.000	0.000	0.000	0.000	0.000	0.000	0.002	0.000	0.000	0.000	0.000	0.000	0.000						
**MF526992/TiLV/Tanzania/TZ2015-01**	0.057	0.065	0.016	0.035	0.033	0.000	0.000	0.000	0.000	0.000	0.000	0.000	0.000	0.000	0.000	0.000	0.000	0.000	0.002	0.000	0.000	0.000	0.000	0.000	0.000	0.000					
**MF526993/TiLV/Tanzania/TZ2015-09**	0.057	0.065	0.016	0.035	0.033	0.000	0.000	0.000	0.000	0.000	0.000	0.000	0.000	0.000	0.000	0.000	0.000	0.000	0.002	0.000	0.000	0.000	0.000	0.000	0.000	0.000	0.000				
**MF526994/TiLV/Tanzania/TZ2015-11**	0.057	0.065	0.016	0.035	0.033	0.000	0.000	0.000	0.000	0.000	0.000	0.000	0.000	0.000	0.000	0.000	0.000	0.000	0.002	0.000	0.000	0.000	0.000	0.000	0.000	0.000	0.000	0.000			
**MF526995/TiLV/Tanzania/TZ2015-14**	0.057	0.065	0.016	0.035	0.033	0.000	0.000	0.000	0.000	0.000	0.000	0.000	0.000	0.000	0.000	0.000	0.000	0.000	0.002	0.000	0.000	0.000	0.000	0.000	0.000	0.000	0.000	0.000	0.000		
**MF526996/TiLV/Tanzania/TZ2015-15**	0.057	0.065	0.016	0.035	0.033	0.000	0.000	0.000	0.000	0.000	0.000	0.000	0.000	0.000	0.000	0.000	0.000	0.000	0.002	0.000	0.000	0.000	0.000	0.000	0.000	0.000	0.000	0.000	0.000	0.000	

Table S6: Pairwise amino acid sequence distances between the Egyptian Amnoonvirus isolates (this study) and partial sequences of Israel and Africa (Lake of Victoria) isolates by PB2 gene. Distances were calculated using the p-distance model. Lower values indicate higher sequence similarity.

	AmnoonvirusEGY1F	AmnoonvirusEGY1H	KU751815/TiLV/Israel/Til-4-2011/Index_Strain	OP037899/TiLV/Israel/939-9/2018	OP037909/TiLV/Israel/939-16/2018	MF536423/TiLV/Uganda/UG2016-01	MF536424/TiLV/Uganda/UG2016-06	MF536425/TiLV/Uganda/UG2016-07	MF536426/TiLV/Uganda/UG2016-04	MF536427/TiLV/Uganda/UG2016-05	MF536428/TiLV/Uganda/UG2016-08	MF536429/TiLV/Uganda/UG2016-02	MF536430/TiLV/Uganda/UG2016-09	MF536431/TiLV/Uganda/UG2016-10	MF526980/TiLV/Tanzania/TZ2015-05	MF526981/TiLV/Tanzania/TZ2015-08	MF526982/TiLV/Tanzania/TZ2015-06	MF526983/TiLV/Tanzania/TZ2015-10	MF526984/TiLV/Tanzania/TZ2015-12	MF526985/TiLV/Tanzania/TZ2015-13	MF526986/TiLV/Tanzania/TZ2015-17	MF526987/TiLV/Tanzania/TZ2015-03	MF526988/TiLV/Tanzania/TZ2015-02	MF526989/TiLV/Tanzania/TZ2015-04	MF526990/TiLV/Tanzania/TZ2015-16	MF526991/TiLV/Tanzania/TZ2015-07	MF526992/TiLV/Tanzania/TZ2015-01	MF526993/TiLV/Tanzania/TZ2015-09	MF526994/TiLV/Tanzania/TZ2015-11	MF526995/TiLV/Tanzania/TZ2015-14	MF526996/TiLV/Tanzania/TZ2015-15
AmnoonvirusEGY1F																															
AmnoonvirusEGY1H	0.068																														
KU751815/TiLV/Israel/Til-4-2011/Index_Strain	0.149	0.169																													
OP037899/TiLV/Israel/939-9/2018	0.176	0.203	0.128																												
OP037909/TiLV/Israel/939-16/2018	0.184	0.211	0.136	0.048																											
MF536423/TiLV/Uganda/UG2016-01	0.149	0.176	0.054	0.101	0.095																										
MF536424/TiLV/Uganda/UG2016-06	0.149	0.176	0.054	0.101	0.095	0.000																									
MF536425/TiLV/Uganda/UG2016-07	0.149	0.176	0.054	0.101	0.095	0.000	0.000																								
MF536426/TiLV/Uganda/UG2016-04	0.149	0.176	0.054	0.101	0.095	0.000	0.000	0.000																							
MF536427/TiLV/Uganda/UG2016-05	0.149	0.176	0.054	0.101	0.095	0.000	0.000	0.000	0.000																						
MF536428/TiLV/Uganda/UG2016-08	0.149	0.176	0.054	0.101	0.095	0.000	0.000	0.000	0.000	0.000																					
MF536429/TiLV/Uganda/UG2016-02	0.149	0.176	0.054	0.101	0.095	0.000	0.000	0.000	0.000	0.000	0.000																				
MF536430/TiLV/Uganda/UG2016-09	0.149	0.176	0.054	0.101	0.095	0.000	0.000	0.000	0.000	0.000	0.000	0.000																			
MF536431/TiLV/Uganda/UG2016-10	0.149	0.176	0.054	0.101	0.095	0.000	0.000	0.000	0.000	0.000	0.000	0.000	0.000																		
MF526980/TiLV/Tanzania/TZ2015-05	0.149	0.176	0.054	0.101	0.095	0.000	0.000	0.000	0.000	0.000	0.000	0.000	0.000	0.000																	
MF526981/TiLV/Tanzania/TZ2015-08	0.149	0.176	0.054	0.101	0.095	0.000	0.000	0.000	0.000	0.000	0.000	0.000	0.000	0.000	0.000																
MF526982/TiLV/Tanzania/TZ2015-06	0.149	0.176	0.054	0.101	0.095	0.000	0.000	0.000	0.000	0.000	0.000	0.000	0.000	0.000	0.000	0.000															
MF526983/TiLV/Tanzania/TZ2015-10	0.149	0.176	0.054	0.101	0.095	0.000	0.000	0.000	0.000	0.000	0.000	0.000	0.000	0.000	0.000	0.000	0.000														
MF526984/TiLV/Tanzania/TZ2015-12	0.155	0.182	0.061	0.108	0.102	0.007	0.007	0.007	0.007	0.007	0.007	0.007	0.007	0.007	0.007	0.007	0.007	0.007													
MF526985/TiLV/Tanzania/TZ2015-13	0.149	0.176	0.054	0.101	0.095	0.000	0.000	0.000	0.000	0.000	0.000	0.000	0.000	0.000	0.000	0.000	0.000	0.000	0.007												
MF526986/TiLV/Tanzania/TZ2015-17	0.149	0.176	0.054	0.101	0.095	0.000	0.000	0.000	0.000	0.000	0.000	0.000	0.000	0.000	0.000	0.000	0.000	0.000	0.007	0.000											
MF526987/TiLV/Tanzania/TZ2015-03	0.149	0.176	0.054	0.101	0.095	0.000	0.000	0.000	0.000	0.000	0.000	0.000	0.000	0.000	0.000	0.000	0.000	0.000	0.007	0.000	0.000										
MF526988/TiLV/Tanzania/TZ2015-02	0.149	0.176	0.054	0.101	0.095	0.000	0.000	0.000	0.000	0.000	0.000	0.000	0.000	0.000	0.000	0.000	0.000	0.000	0.007	0.000	0.000	0.000									
MF526989/TiLV/Tanzania/TZ2015-04	0.149	0.176	0.054	0.101	0.095	0.000	0.000	0.000	0.000	0.000	0.000	0.000	0.000	0.000	0.000	0.000	0.000	0.000	0.007	0.000	0.000	0.000	0.000								
MF526990/TiLV/Tanzania/TZ2015-16	0.149	0.176	0.054	0.101	0.095	0.000	0.000	0.000	0.000	0.000	0.000	0.000	0.000	0.000	0.000	0.000	0.000	0.000	0.007	0.000	0.000	0.000	0.000	0.000							
MF526991/TiLV/Tanzania/TZ2015-07	0.149	0.176	0.054	0.101	0.095	0.000	0.000	0.000	0.000	0.000	0.000	0.000	0.000	0.000	0.000	0.000	0.000	0.000	0.007	0.000	0.000	0.000	0.000	0.000	0.000						
MF526992/TiLV/Tanzania/TZ2015-01	0.149	0.176	0.054	0.101	0.095	0.000	0.000	0.000	0.000	0.000	0.000	0.000	0.000	0.000	0.000	0.000	0.000	0.000	0.007	0.000	0.000	0.000	0.000	0.000	0.000	0.000					
MF526993/TiLV/Tanzania/TZ2015-09	0.149	0.176	0.054	0.101	0.095	0.000	0.000	0.000	0.000	0.000	0.000	0.000	0.000	0.000	0.000	0.000	0.000	0.000	0.007	0.000	0.000	0.000	0.000	0.000	0.000	0.000	0.000				
MF526994/TiLV/Tanzania/TZ2015-11	0.149	0.176	0.054	0.101	0.095	0.000	0.000	0.000	0.000	0.000	0.000	0.000	0.000	0.000	0.000	0.000	0.000	0.000	0.007	0.000	0.000	0.000	0.000	0.000	0.000	0.000	0.000	0.000			

**MF526995/TiLV/Tanzania/TZ2015-14**	**0.149**	**0.176**	**0.054**	**0.101**	**0.095**	**0.000**	**0.000**	**0.000**	**0.000**	**0.000**	**0.000**	**0.000**	**0.000**	**0.000**	**0.000**	**0.000**	**0.000**	**0.000**	**0.007**	**0.000**	**0.000**	**0.000**	**0.000**	**0.000**	**0.000**	**0.000**	**0.000**	**0.000**	**0.000**	
**MF526996/TiLV/Tanzania/TZ2015-15**	**0.149**	**0.176**	**0.054**	**0.101**	**0.095**	**0.000**	**0.000**	**0.000**	**0.000**	**0.000**	**0.000**	**0.000**	**0.000**	**0.000**	**0.000**	**0.000**	**0.000**	**0.000**	**0.007**	**0.000**	**0.000**	**0.000**	**0.000**	**0.000**	**0.000**	**0.000**	**0.000**	**0.000**	**0.000**	**0.000**
